# Plasma Exosomal miRNA Expression Profile as Oxaliplatin-Based Chemoresistant Biomarkers in Colorectal Adenocarcinoma

**DOI:** 10.3389/fonc.2020.01495

**Published:** 2020-09-18

**Authors:** Jiayi Han, Wu Sun, Rui Liu, Zhen Zhou, Haiyang Zhang, Xi Chen, Yi Ba

**Affiliations:** ^1^Key Laboratory of Cancer Prevention and Therapy, National Clinical Research Center for Cancer, Tianjin's Clinical Research Center for Cancer, Tianjin Medical University Cancer Institute and Hospital, Tianjin Medical University, Tianjin, China; ^2^The Comprehensive Cancer Centre of Drum Tower Hospital, Medical School of Nanjing University and Clinical Cancer Institute of Nanjing University, Nanjing, China; ^3^State Key Laboratory of Pharmaceutical Biotechnology, Collaborative Innovation Center of Chemistry for Life Sciences, Jiangsu Engineering Research Center for MicroRNA Biology and Biotechnology, School of Life Sciences, Nanjing University Advanced Institute for Life Sciences (NAILS), Nanjing University, Nanjing, China

**Keywords:** circulating biomarkers, miRNA profile, exosome, chemoresistance, colorectal cancer

## Abstract

**Background:** Chemotherapy is one of the most common therapies used in the treatment of colorectal cancer (CRC), but chemoresistance inevitably occurs. It is challenging to obtain an immediate and accurate diagnosis of chemoresistance. The potential of circulating exosomal miRNAs as oxaliplatin-based chemoresistant biomarkers in CRC patients was investigated in this study.

**Methods:** Plasma exosomal miRNAs in sensitive and resistant patients were analyzed by miRNA microarray analysis, followed by verification with a quantitative reverse-transcription polymerase chain reaction (RT-qPCR) assay in two independent cohorts. The diagnostic accuracy was determined by ROC curve analysis. Logistic regression analysis and Spearman's rank correlation test were also performed. Finally, bioinformatics was used to preliminarily explore the potential molecular mechanism of the selected miRNAs in chemoresistance.

**Results:** miRNA microarray analysis identified four upregulated miRNAs and 20 downregulated miRNAs in chemoresistant patients compared to chemosensitive patients. Twelve markedly dysregulated miRNAs were selected for further investigation, of which six (miR-100, miR-92a, miR-16, miR-30e, miR-144-5p, and let-7i) were verified to be significantly and consistently dysregulated (>1.5-fold, *P* < 0.05). The combination of the six miRNAs had the highest AUC (0.825, 95% CI, 0.753–0.897). The expression level of these 6 miRNAs was not correlated with tumor location, stage, or chemotherapy program. Only miR-100 was significantly upregulated in low histological grade. GO analysis and KEGG pathway analysis showed that miRNAs were related to RNA polymerase II transcription and enriched in the PI3K-AKT signaling pathway, AMPK signaling pathway, and FoxO signaling pathway.

**Conclusions:** We identified a panel of plasma exosomal miRNAs, containing miR-100, miR-92a, miR-16, miR-30e, miR-144-5p, and let-7i, that could significantly distinguish chemoresistant patients from chemosensitive patients. The detection of circulating exosomal miRNAs may serve as an effective way to monitor CRC patient responses to chemotherapy. Targeting these miRNAs may also be a promising strategy for CRC treatment.

## Introduction

Colorectal cancer (CRC) is one of the most frequently diagnosed cancers worldwide, with high morbidity and mortality rates. In 2019, there were ~1.4 million estimated new cases and 51 thousand estimated deaths in the USA (1). CRC contributes to ~10% of all cancer cases worldwide (2). Among all therapy methods, chemotherapy is of great importance in prolonging the survival of CRC patients. However, only ~50% of CRC patients respond to first-line chemotherapy (3). Doctors need to monitor patient responses during chemotherapy and change the therapeutic regimen immediately when chemoresistance occurs to maximize patient benefit from chemotherapy. However, there is currently no sensitive method to detect tumor resistance in the clinic. Blood-based tests have distinct advantages in that they are minimally invasive and easy to acquire. We aimed to identify novel biomarkers to detect, monitor, and predict CRC patient responses to chemotherapy.

MicroRNAs (miRNAs) are evolutionarily conserved non-coding RNAs, with a length of 18–22 nt. miRNAs have been proven to play critical roles in most biological processes, including cancers (4, 5). Previous research in our laboratory showed that miRNAs can stably exist in circulating blood and may serve as blood-based biomarkers (6). Secreted miRNAs, especially those in exosomes, can mediate communication between different tissues and thus modulate distant cell functions and gene expression (7, 8). Researchers have found that exosomal miRNAs confer chemoresistance in various cancers (9–11), including colorectal cancer (12). However, there is no systematic research about the diagnostic value of plasma exosomal miRNAs in chemoresistance in CRC. Exosomal miRNAs have natural advantages due to their stable structure and minimal non-specific interference (7) and may serve as better biomarkers to detect chemoresistance in CRC.

In this study, we utilized miRNA microarray analysis followed by verification with RT-qPCR assay to identify aberrantly expressed plasma exosomal miRNAs in responsive and resistant CRC patients receiving oxaliplatin-based chemotherapy. We then assessed the diagnostic value of these miRNAs as promising biomarkers for chemoresistance. Our results demonstrate that plasma exosomal miRNAs can serve as effective markers to monitor the chemotherapy response of CRC patients.

## Materials and Methods

### Participants and Sample Collection

In the present study, we collected plasma samples from 210 late-stage CRC patients (III-IV) at Tianjin Medical University Cancer Institute and Hospital between May 2015 and December 2017. We obtained informed consent from every enrolled patient, and the protocols were approved by the Ethics Committee of Tianjin Medical University Cancer Institute and Hospital. All patients were diagnosed with colorectal cancer by endoscopic biopsy and identified as inoperable. They did not previously have any other type of tumor nor had they received any chemotherapy. Of all the samples, 47 were collected before chemotherapy, and the rest were obtained immediately when the patients were diagnosed as chemoresistant (PD for resistance group) or chemosensitive (PR for response group) after they received oxaliplatin-based chemotherapy (FOLFOX or XELOX). None of the patients received any targeted therapy. PD or PR was defined according to the RECIST 1.1 criteria (13). Chemoresistant patients (*n* = 79) were defined as having new distant metastases or an enlarged tumor volume, while chemosensitive patients (*n* = 84) were defined as having significant tumor shrinkage. All of the imaging diagnostics were independently evaluated by three doctors and two radiologists. All peripheral blood samples were centrifuged at 1,500 rpm for 10 min at 4°C, and the supernatants were collected and stored at −80°C for further use.

### Isolation of Exosomes and Extraction of RNA

The plasma samples were fully thawed and then centrifuged in three steps (300 g for 10 min, 2,000 g for 20 min, and 10,000 g for 20 min) to remove any cells and cell debris. Exosome isolation was carried out by the Total Exosome Isolation Kit (from plasma) (Invitrogen, MA, USA) according to the manufacturer's instructions. A 200 μl plasma sample was mixed with 100 μL 1 × PBS, and then 60 μL Exosome Precipitation Reagent (from plasma) was added. The thoroughly mixed mixture was incubated at room temperature for 10 min and then centrifuged at 10,000 g for 5 min. The supernatant was discarded, and 100 μL of 1 × PBS was added to the pellet to fully resuspend the exosomes. Then, the exosomes were used to extract RNA or for other analyses.

The miRNeasy Kit (Qiagen, Hilden, Germany) was used to extract the RNA from resuspended exosomes according to the manufacturer's instructions. Finally, total RNA was dissolved in 20 μL RNase-free water, quantified by NanoDrop 2,000 (Thermo, MA, USA), and stored at −80°C until further analysis.

### Nanoparticle Tracking Analysis (NTA)

One hundred microliters of resuspended exosomes were diluted in 1 mL of 1 × PBS and then measured by a NanoSight NS 300 system (NanoSight Technology, Malvern, UK) following our previously described procedure (14). NTA analytical software (version 2.3) was used to analyze the data.

### Transmission Electron Microscopy (TEM) and Western Blotting

The TEM assay was performed as we previously described (15). The suspended exosomes were placed onto a carbon-coated 200-mesh copper grid for 20 min. The main yield was added to 2% phosphotungstic acid solution (HT152, Sigma, Germany) for negative staining for 10 min at an ambient temperature. The copper grids were carefully wiped with filter paper to remove any excess liquid and then dried with an incandescent lamp. A JEM-1011 scanning transmission electron microscope (Hitachi, Tokyo, Japan) was used for further photomicrography.

Total exosomal protein was extracted by RIPA buffer for subsequent western blotting assay. The proteins were separated according to their molecular weight by a 10% SDS-PAGE gel and then transferred to a polyvinylidene difluoride (PVDF) membrane. The PVDF membrane was blocked with 5% skimmed milk for at least 1 h at room temperature and incubated with primary antibodies at 4°C overnight. The primary antibodies we used were as follows: anti-CD9 (1:1000, Proteintech), anti-Alix (1:1000, Proteintech), and anti-TSG101 (1:1000, Proteintech). After incubating with secondary antibodies for 1 h at room temperature, the PVDF membrane was then added to ECL western blotting substrate (Thermo Fisher Scientific, MA, USA) to obtain a clear band on the gel imaging system.

### Quantification of miRNAs

A probe-based RT-qPCR assay was used to detect the levels of exosomal miRNAs in individual plasma samples as previously reported (15). In the present study, we normalized the plasma exosome miRNA level to the plasma volume, as the candidate endogenous controls [miR-16 (16) and let-7i (17)] were changed in our study. Cel-miR-39 was added as an external control according to the manufacturer's instructions (miRNeasy Kit, Qiagen). The relative levels of miRNAs were normalized to Cel-miR-39 and calculated as 2^−ΔCt^, where ΔCt was calculated as (Ct value of candidate miRNA)–(Ct value of Cel-miR-39). The details of the RT-qPCR system are shown in the Methods section in the Supplementary Data.

### Gene Ontology (GO) and Kyoto Encyclopedia of Genes and Genomes (KEGG) Pathways

GO and KEGG pathway analyses were performed as previously described (18). More details are provided in the Supplementary Data.

### Data Analysis

All statistical analyses were performed using IBM SPSS Statistics (version 25) and graphed by GraphPad Prism (version 7.00). Data are presented as the means ± SDs for the miRNAs. Two-group analysis was performed by Student's *t*-test while three-group analysis was performed by one-way ANOVA. *P* < 0.05 was considered statistically significant. Receiver operating characteristic (ROC) curves and areas under the ROC curves (AUCs) were separately generated and calculated to estimate the diagnostic value of the candidate exosomal miRNAs on chemoresistance in CRC. We calculated the risk score to evaluate the potential of the combination of the candidate miRNAs to diagnose chemoresistance in CRC as previously described (19). The detailed procedure of the risk score analysis is shown in Methods in the Supplementary Data. Binary logistic regression analysis was performed to analyze potential risk factors, and Spearman's rank correlations were performed to verify the correlations of selected miRNAs with clinical parameters.

## Results

### Characterization of the Plasma Exosomes

**Figure 1:**
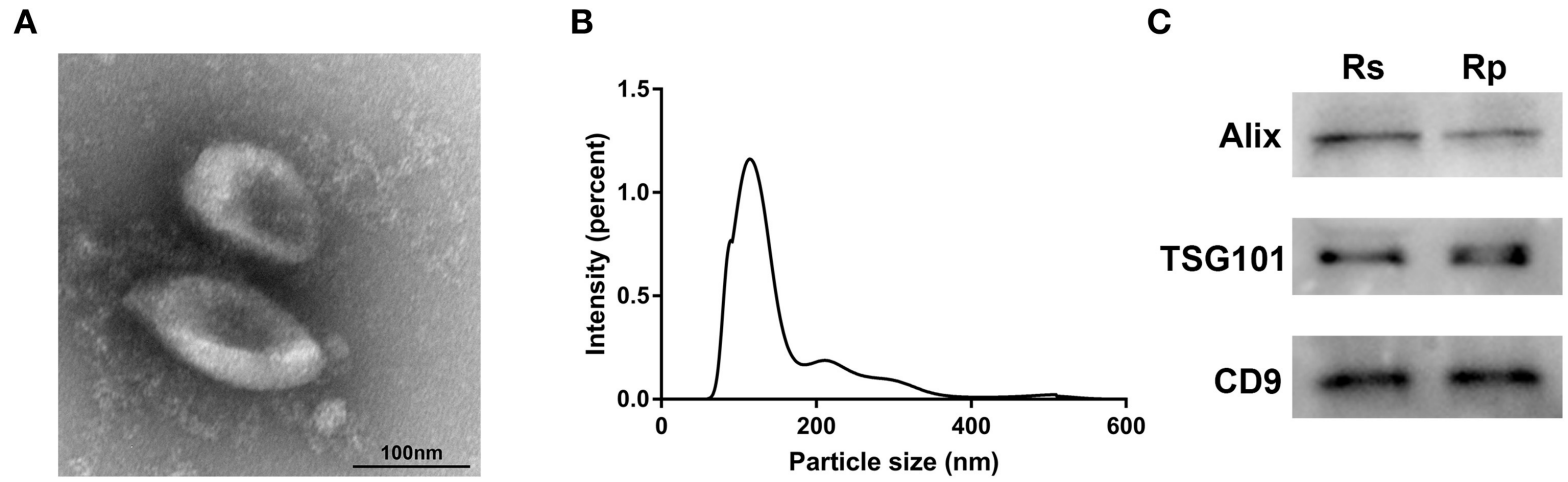
Characterization of exosomes derived from the plasma of chemoresistant and chemosensitive CRC patients. **(A)** The morphology and size of exosomes by TEM. **(B)** The size distribution of exosomes, analyzed by NTA. **(C)** Western blots of the exosomal markers: CD9, Alix, and TSG101. Rs, Resistance; Rp, Response.

To identify the quality and purity of the exosomes we extracted from the plasma, we first visualized these particles by TEM and observed membrane-bound spherical structures of ~100 nm (Figure 1A), which are typical characteristics of exosomes. NTA analysis was then performed to assess the size distribution of the exosomes. As shown in Figure 1B, most of the particles we extracted were ~100 nm in diameter (Supplementary Table 1). Western blotting analysis showed that the known exosomal markers (CD9, TSG101, and Alix) were markedly detectable in our extracted particles (Figure 1C). These results demonstrate that we successfully extracted exosomes from plasma.

### Screening of Plasma Exosomal miRNAs for Chemoresistance Diagnosis

**Figure 2:**
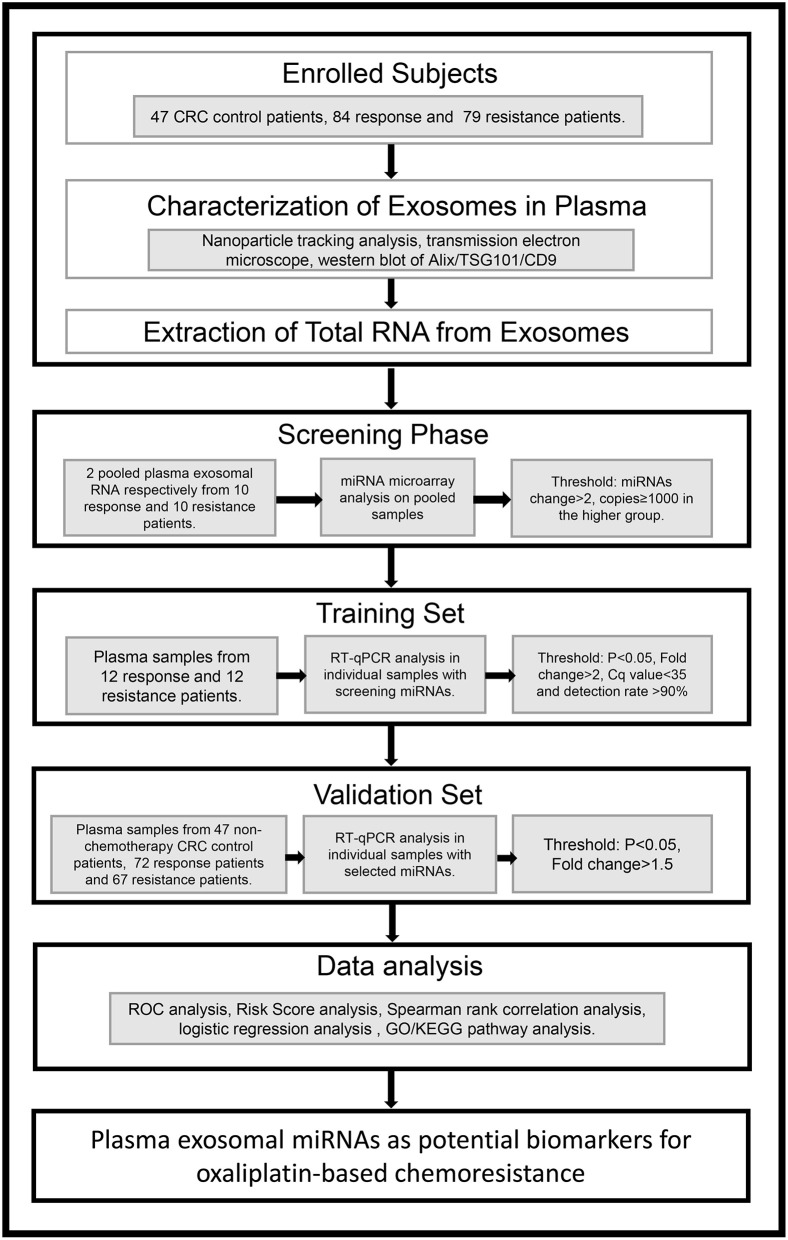
An overview of the experimental design.

**Figure 3:**
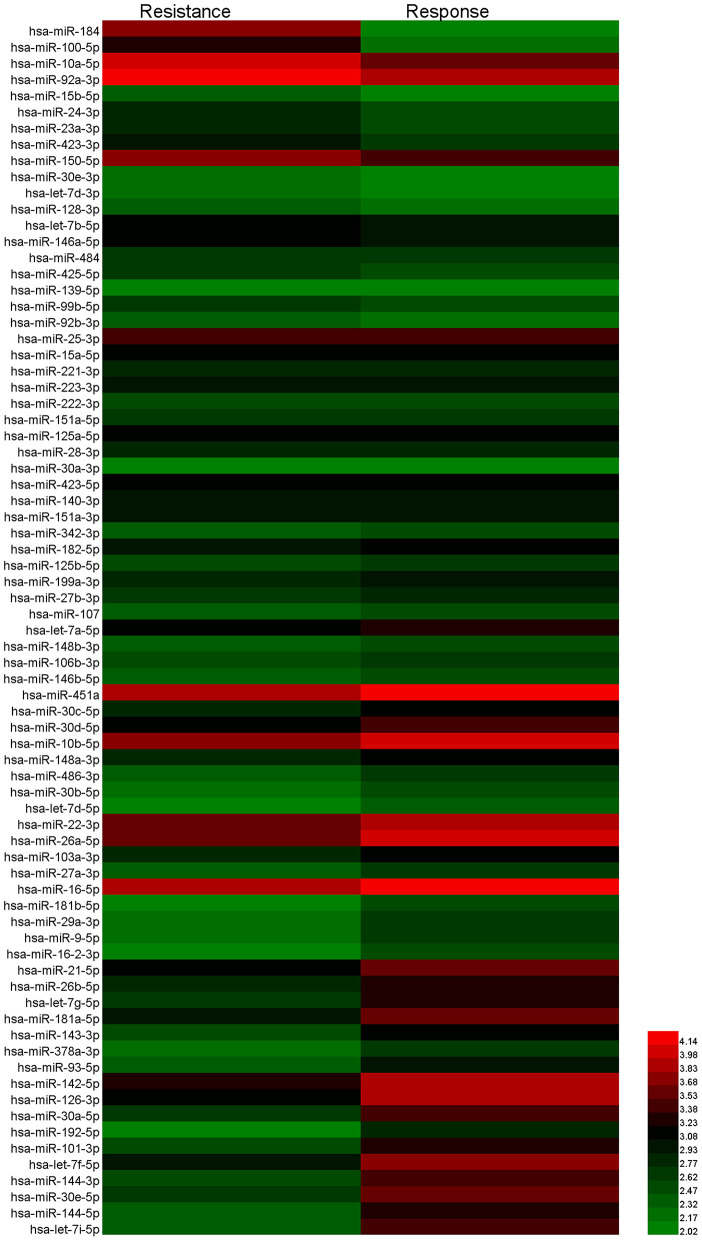
Heat map showing the significantly upregulated and downregulated miRNAs in the resistance group compared to the response group.

To identify the candidate miRNAs that change during chemoresistance and to evaluate the diagnostic value of the chosen miRNAs, we designed a multiphase study as shown in Figure 2. We first performed a miRNA microarray analysis to screen the candidate miRNAs and measured 646 miRNAs in pooled plasma exosome samples from the response group and resistance group (each pooled from 10 individuals). The highly-expressed miRNAs between the two groups are shown in the heat map (Figure 3 and Supplementary Table 2). In this study, we considered miRNAs to be markedly changed if the higher group reads were >1,000, and fold change was ≥2. Consequently, four upregulated miRNAs (Supplementary Table 3) and 20 downregulated miRNAs (Supplementary Table 4) were identified in our study. All upregulated miRNAs and the top seven downregulated miRNAs with a fold change <0.2 were selected for validation. As miR-16 was considered as an internal control in several previous plasma miRNA examinations but its expression did change in our miRNA microarray analysis, we also added miR-16 for further validation.

### Validation of the Candidate miRNAs by RT-qPCR

To confirm the results of the miRNA microarray analysis, we then performed RT-qPCR in three independent cohorts (47 CRC control patients, 84 responsive patients, and 79 resistant patients. The quantification of RNA was shown in Supplementary Table 5). First, we detected the expression level of the 12 candidate miRNAs in 12 response patients and 12 resistance patients in the training set (Supplementary Table 6). We considered a miRNA to be significantly changed between the two groups if it met the following criteria: *P* < 0.5, fold change >2 or <0.5, quantification cycle (Ct) value <35, and detection rate >75%, as previously described (17). Under these conditions, we ultimately selected eight miRNAs (miR-184, miR-100, miR-10a, miR-92a, let-7i, miR-144-5p, miR-30e, and miR-16) for further analysis (The mature sequence and catalog ID of these miRNAs were shown in Supplementary Table 13).

**Figure 4:**
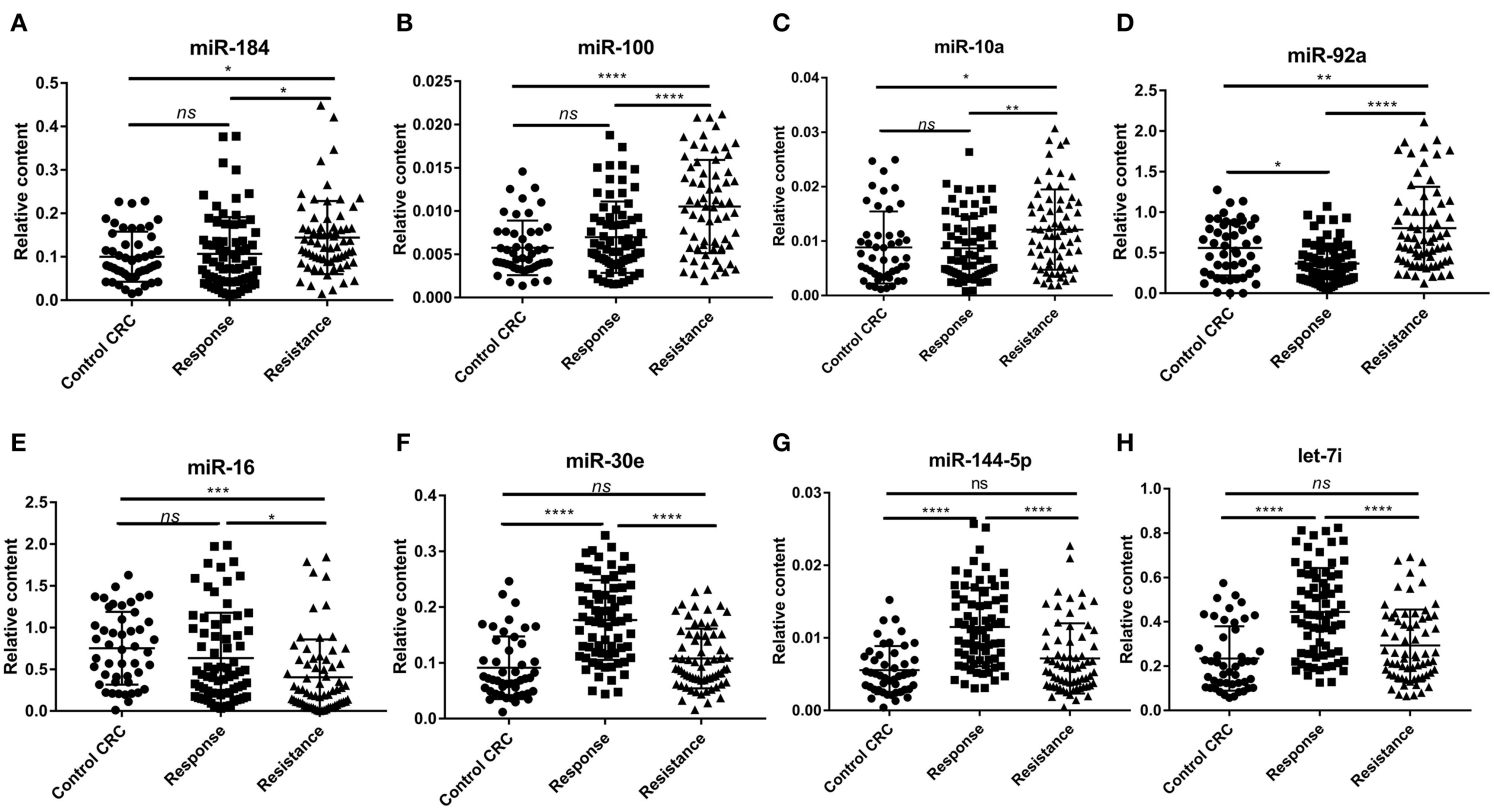
The relative expression of plasma exosomal miRNAs in the control CRC group (*n* = 47), response group (*n* = 72), and resistance group (*n* = 67). **(A)** Relative expression of plasma exosomal miR-184. **(B)** Relative expression of plasma exosomal miR-100. **(C)** Relative expression of plasma exosomal miR-10a. **(D)** Relative expression of plasma exosomal miR-92a. **(E)** Relative expression of plasma exosomal miR-16. **(F)** Relative expression of plasma exosomal miR-30e. **(G)** Relative expression of plasma exosomal miR-144-5p. **(H)** Relative expression of plasma exosomal let-7i. Statistics were analyzed by one-way ANOVA, data was shown as mean ± SD. *P < 0.05, **P < 0.01, ***P < 0.001, ****P < 0.0001.

To confirm the diagnostic value of our selected miRNAs on the chemoresistance of CRC patients, we validated our results in a larger population (72 responsive patients and 67 resistant patients). To identify the exact reason for the dysregulation of the selected miRNAs, we also recruited 47 CRC control patients, who were diagnosed with late-stage CRC without receiving any chemotherapy. Table 1 shows the clinical features of the patients in the validation cohorts. The results are shown in Figure 4. The eight selected miRNAs were significantly different between the response group and the resistance group. miR-184, miR-100, miR-10a, and miR-92a were upregulated in the resistance group compared to the response group, while let-7i, miR-144, miR-30e, and miR-16 showed the opposite regulation. The upregulation of miR-184, miR-100-5p, and miR-10a was due to the higher expression levels in resistant patients, as there was no significant difference between the control CRC group and the response group (Figures 4A–C). miR-92a was not only upregulated in the resistance group but also slightly decreased in the response group compared to the control CRC group (Figure 4D). Let-7i, miR-30e, and miR-144 were upregulated in the response group while there was no marked difference between the control CRC group and the resistance group (Figures 4F–H). The dysregulation of miR-16 was due to its downregulation in the resistance group compared to the control CRC group and response group (Figure 4E). The detailed data of each group are shown in Supplementary Table 7. In this section, miRNAs were regarded as markedly dysregulated between resistant patients and responsive patients if they met the following criteria: fold change ≥1.5 or ≤0.67, *P* < 0.05, Ct value <35, and detection rate >75%. Finally, six miRNAs, miR-100, miR-92a, miR-16, miR-30e, miR-144-5p, and let-7i, were chosen for further analysis.

**Table 1:** Clinical features of the patients in the validation cohorts.

**Variables**	**CRC control (*n* = 47)**	**Response group (*n* = 72)**	**Resistance group (*n* = 67)**	*P*-value^*^
**Age (years)**				
Mean (SD)	60.91 (6.068)	57.88 (9.246)	60.43 (9.767)	0.283
>60, No.	23	37	37	
<60, No.	24	35	30	
**Sex, no**.				0.969
Male	26	40	37	
Female	21	32	30	
**Localization**				0.959
Rectum	28	39	36	
Colon	19	33	31	
**Histological grade, no**.				0.883
Middle-Low	33	43	42	
High	14	29	25	
**TNM stage, no**.				
III	23	31	25	0.49
IV	24	41	42	
**Chemotherapy program**				0.464
FOLFOX		41	34	
XELOX		31	33	
**Clinical evaluation**				
Response		72	0	
Resistance		0	67	

* *Two-sided X^2^-test between response group and resistance group*.

### Diagnostic Accuracy of the Candidate miRNAs

**Figure 5:**
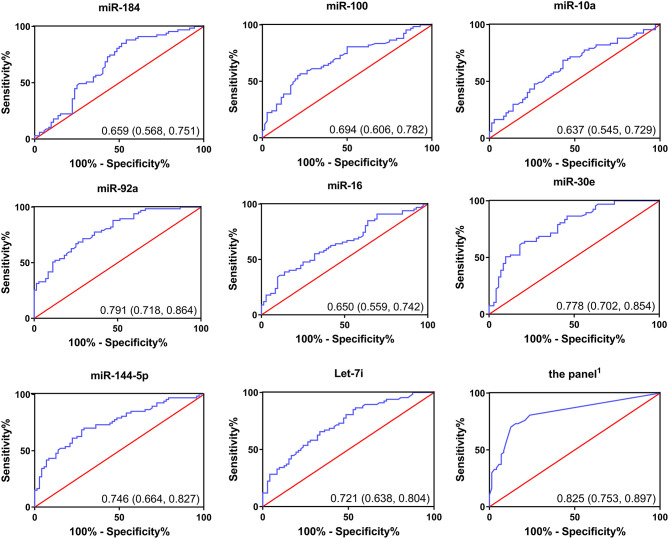
Receiver operating curves (ROC) of selected miRNAs for differentiation between responsive patients and resistant patients based on the plasma exosomal miRNA profile.

We performed ROC curve analysis to evaluate the diagnostic accuracy of the selected miRNAs for chemoresistance in CRC. As shown in Figure 5, the AUCs of the candidate miRNAs ranged from 0.637 to 0.791. Among all of the selected miRNAs, miR-92a had the highest AUC (0.791; 95% CI, 0.718–0.864), followed by miR-30e (0.778; 95% CI, 0.702–0.854). The six miRNAs we selected above had relatively high accuracy. In addition, we calculated the diagnostic accuracy of the combination of these six miRNAs by our previously described method (20). Our results showed that the combined miRNA panel had the highest accuracy, with an AUC of 0.825 (95% CI, 0.753–0.897, Supplementary Table 8). The diagnostic accuracy of CEA and CA19-9 in our research was 0.542 and 0.686, respectively. The combined analysis of these miRNAs and tumor biomarkers did not result in higher accuracy (Supplementary Table 8). Together, these results suggest that the six miRNAs we selected can differentiate between resistant and responsive CRC patients receiving chemotherapy with higher accuracy than traditional tumor biomarkers. The combined detection of these six miRNAs could be a powerful indicator for monitoring chemotherapy effects.

### Binary Logistic Regression Analysis of the Risk Score in Resistant Patients and Responsive Patients

We then performed univariate logistic regression analysis to explore the risk prediction value of the candidate miRNAs in our study. The response/resistance status was defined as the dependent variable and the risk score, which we calculated by the combined diagnostic accuracy analysis and was defined as the covariate. The regression coefficients of all six miRNAs were larger than 0 (ranging from 1.502 to 2.185). The odds ratios were all >1 (ranging from 4.491 to 8.886) (Supplementary Table 9). These results indicate that all six miRNAs are potential risk factors for chemoresistance diagnosis in CRC patients.

### Correlations of Selected Plasma Exosomal miRNAs With Other Clinical Parameters

To further investigate whether other clinical parameters influence the expression of these miRNAs, Spearman's rank correlation test was performed as our next step. As shown in Supplementary Table 10, the expression levels of all six miRNAs were not related to sex, age, tumor location, TNM stage, or chemotherapy program. Only miR-100 was significantly negatively associated with the tumor histological grade (*r* = −0.177, *P* = 0.037). These results demonstrate that the expression levels of candidate miRNAs were not influenced by basic clinical parameters. However, the mechanism of miR-100 on CRC histological grade still needs further study.

### GO and KEGG Pathway Analyses of Candidate miRNA Target Genes

**Figure 6:**
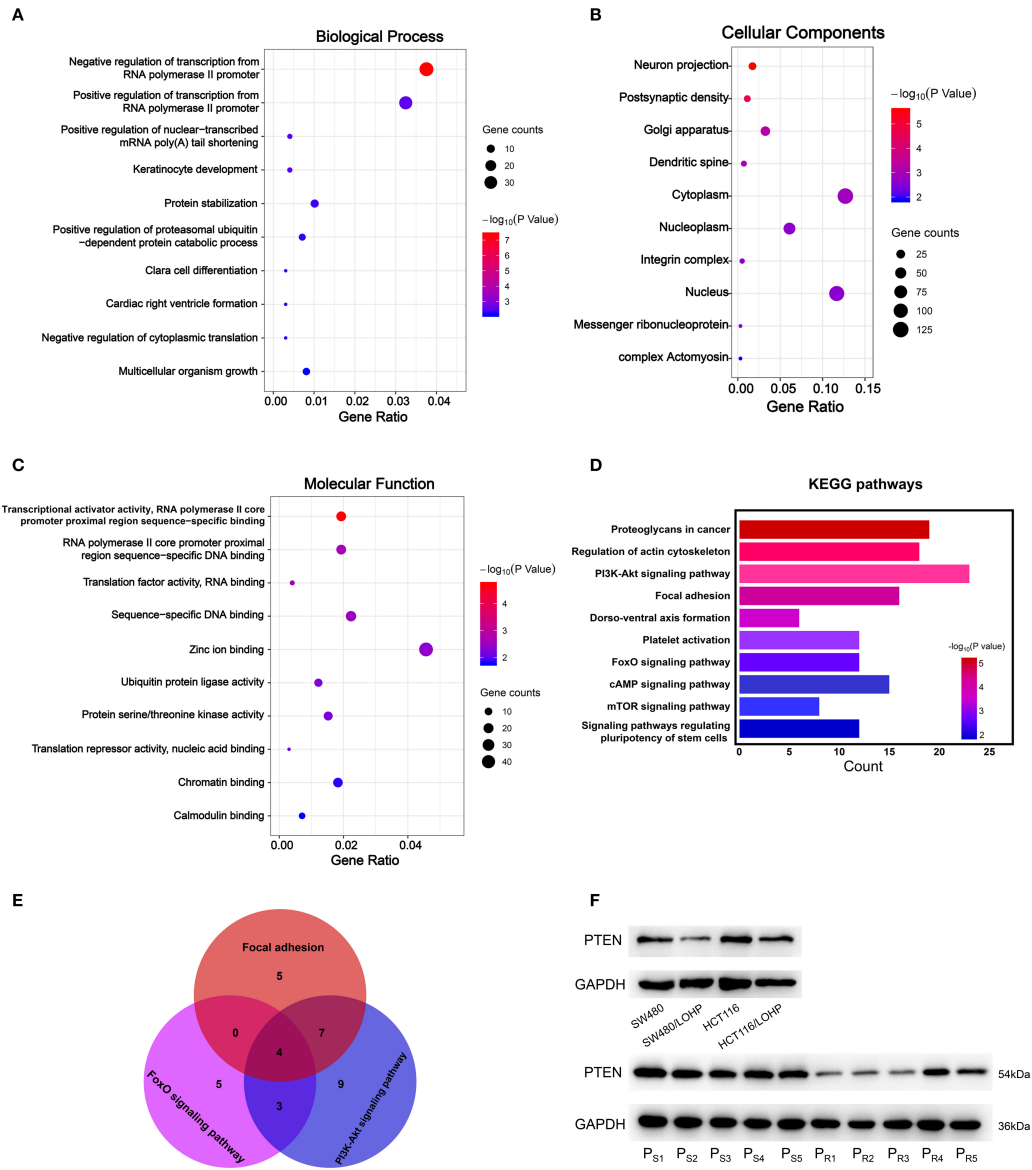
GO and KEGG pathway analyses of downstream target genes of two upregulated miRNAs. **(A-C)**: GO analysis. **(A)** Biological processes. **(B)** Cellular component. **(C)** Molecular function. **(D)** The top ten KEGG pathways enriched in the target genes. **(E)** The analysis of Focal adhesion, FoxO signaling pathway, and PI3K-Akt signaling pathway. **(F)** The identified target gene PTEN was measured by Western blot in CRC cell lines and tissues. P_S_: chemosensitive CRC patients. P_R_: chemoresistant CRC patients.

**Figure 7:**
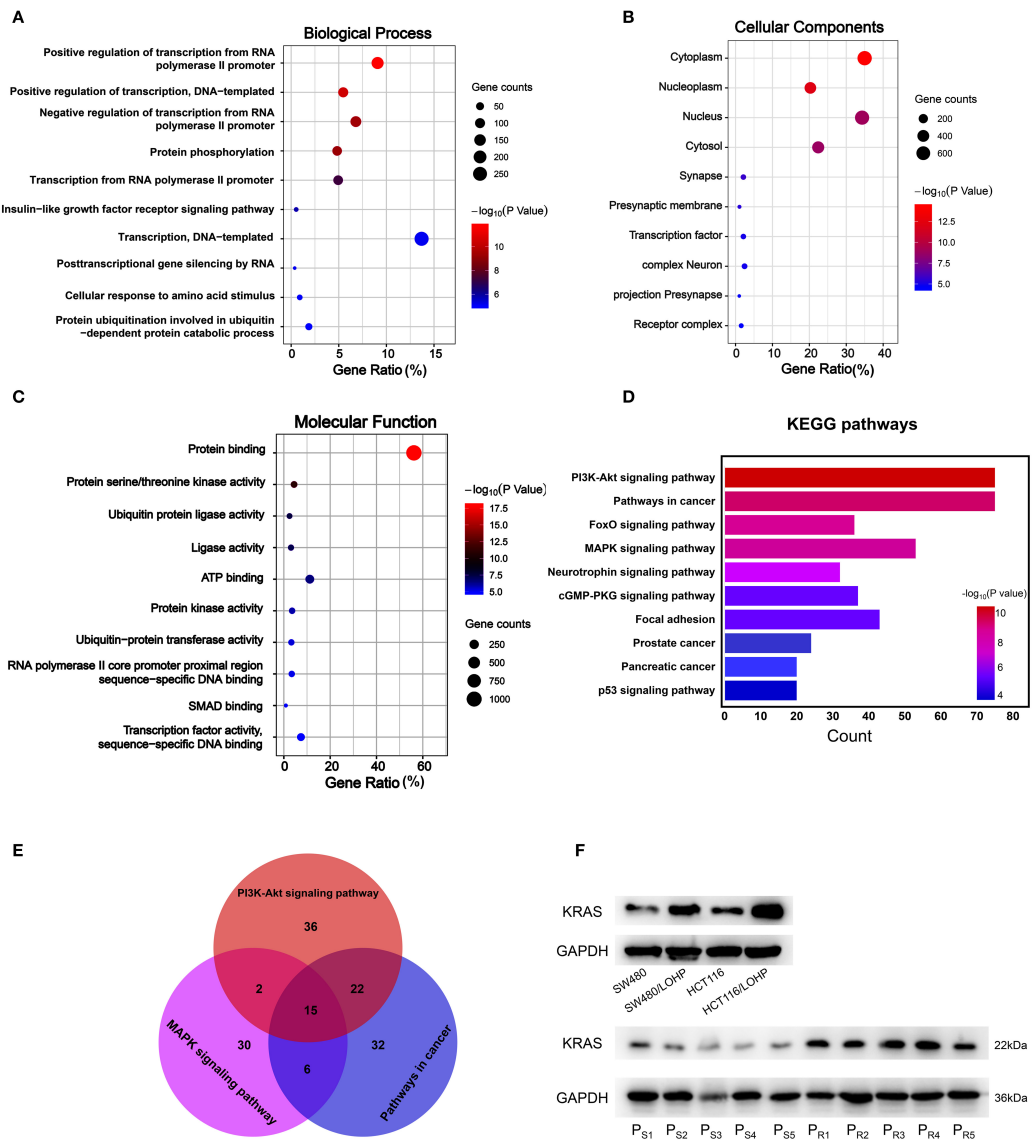
GO and KEGG pathway analyses of downstream target genes of four downregulated miRNAs. **(A-C)**: GO analysis. **(A)** Biological processes. **(B)** Cellular component. **(C)** Molecular function. **(D)** The top ten KEGG pathways enriched in the target genes. **(E)** The analysis of PI3K-Akt signaling pathway, MAPK signaling pathway, and pathways in cancer. **(F)** The identified target gene KRAS was measured by Western blot in CRC cell lines and tissues. P_S_: chemosensitive CRC patients. P_R_: chemoresistant CRC patients.

Next, we explored the potential biological roles of the candidate miRNAs in chemoresistance. The potential target genes were predicted by three bioinformatics tools (TargetScan, miRDB, and microT-CDS). We first performed Gene Ontology analysis for three categories: biological processes (BPs), cellular components (CCs), and molecular functions (MFs). Figures 6, 7 Show the analysis of two upregulated and four downregulated miRNAs in chemoresistance separately. The downstream target genes of upregulated miRNAs were mainly enriched in the negative regulation of transcription from RNA polymerase II promoter (Figure 6A), while the target genes of downregulated miRNAs were mainly enriched in positive regulation of transcription from RNA polymerase II promoter (Figure 7A), indicating that RNA polymerase II may play a critical role in chemoresistance in CRC. In addition, protein stabilization (Figure 6A, *P* < 0.001) and protein phosphorylation (Figure 7A, *P* < 0.001) may also have a significant effect on chemoresistance in CRC. Among cellular component (CC) terms, these genes were mainly distributed in the synapses and Golgi apparatus (Figures 6B, 7B, *P* < 0.001), which means that signal transduction and membrane formation may influence chemoresistance. The top molecular functions (MFs) were zinc ion binding (Figure 6C, *P* < 0.01) and protein binding (Figure 7C). Furthermore, the top ten pathways identified by KEGG pathway analysis are shown in Figures 6D, 7D (*P* < 0.001). The PI3K-AKT signaling pathway, cAMP signaling pathway, FoxO signaling pathway, and MAPK signaling pathway accounted for most of the identified pathways, all of which have been reported to be related to chemoresistance (21–23). We overlapped the first three signaling pathways and obtained four genes in the upregulated group and 15 genes in the downregulated group that could be coregulated by our selected miRNAs (Figures 6E, 7E and Supplementary Tables 11, 12). Among all predicted genes, we observed PTEN and KRAS, which have been reported to be chemoresistance-related regulators (24, 25), were predicted to be dysregulated in chemoresistant CRC patients. To confirm our predicted genes, we further measured the expression levels of PTEN and KRAS in chemoresistant cells and tissues. As shown in Figures 6F, 7F, PTEN was downregulated while KRAS was up-regulated in both chemoresistant cell lines and tissues compared to their parental cell lines and chemosensitive tissues.

## Discussion

Previous studies have demonstrated that the expression of circulating exosomal miRNAs can change during CRC development as well as after receiving chemotherapy (26). There are also studies reporting that serum exosomal miRNAs may predict the response of CRC patients to chemotherapy (18). However, few studies have evaluated the difference in miRNA expression levels between responsive patients and resistant patients after receiving chemotherapy in CRC. Currently, clinical doctors have to test tumor markers, such as CA199 and CEA, or regularly perform a CT scan to monitor patient responses to chemotherapy, which are imprecise, insensitive, and time-consuming methods. In the present study, we designed a multiphase, case-control study to identify the differentially expressed miRNAs in circulating exosomes between the chemosensitive patients and the chemoresistant patients receiving oxaliplatin-based chemotherapy, aiming to provide a sensitive and effective method to distinguish tumor-resistant patients in a timely manner, guide the selection of chemotherapy regimens, and improve the prognosis of CRC patients.

First, we performed a miRNA microarray analysis and identified four upregulated miRNAs and 20 downregulated miRNAs in chemoresistant patients compared to chemosensitive patients. After verifying the results in a large cohort of CRC patients, we found that the miRNA expression profile in the chemoresistant patients was markedly different from that of the chemosensitive patients. We identified eight miRNAs, miR-184, miR-100, miR-10a, miR-92a, miR-16, miR-30e, miR-144-5p, and let-7i, that were significantly and consistently differentially expressed between these two groups. The AUCs of these miRNAs for CRC chemoresistance diagnosis varied from 0.637 to 0.791. We selected six of these miRNAs (except for miR-10a and miR-184) with a fold change over 1.5 for combination analysis of their diagnostic accuracy. The AUC of this panel was 0.825, which was higher than any of the single miRNAs. Moreover, the diagnostic accuracy of the CRC biomarkers CEA and CA19-9 on the progression of CRC is 0.542 and 0.686, respectively, which were consistent with previous reports (27). Our research provides a more valuable plasma biomarker for evaluating CRC dynamics. In addition, the combined analysis of this panel of miRNAs and CRC biomarkers did not show a higher diagnostic accuracy. Moreover, the binary logistic regression analysis indicated that all six miRNAs are potential risk factors for chemoresistance diagnosis in CRC patients. We also found that only miR-100 had a slight difference between different tumor histological grades. Whether there is physiological significance and what the specific mechanism is still need further study.

miR-16 is usually used as an internal control due to its stable expression level in most cases, including in colorectal cancer (17, 28). The expression level of miR-16 in CRC tissues was also complicated due to its upregulation (29) or downregulation (30). However, none of these studies investigated the expression level of miR-16 in circulating exosomes. It has been reported that the expression level of miR-16 is negatively related to the CSC phenotype (29), and higher expression of miR-16 could reverse chemoresistance by inhibiting CSC properties (31). More studies revealed that the overexpression of miR-16 could sensitize cancer cells to chemotherapy by targeting ATG4B (32), BCL2 (33), CCNJ, or FUBP1 (34). Additionally, it has been reported that exosomal miR-16 could be secreted by CRC tissues into the circulatory system (35). Therefore, it is reasonable to infer that exosomal miR-16 may transfer chemoresistance among CRC cells. Other miRNAs we selected, such as miR-92a (36), miR-100 (37), Let-7i (38), miR-30e (39), and miR-144-5p (40), were all reported to regulate chemoresistance in human cancers and were demonstrated to be enriched in circulating exosomes (41–44).

To further investigate which biological function was influenced by our selected miRNAs in chemoresistance, we utilized bioinformatics tools and performed GO analysis and KEGG pathway analysis. Interestingly, our results showed that the regulation of transcription from the RNA polymerase II promoter may be associated with one of the most prominent differences in chemoresistance in CRC. Researchers have found that RNA polymerase could modulate drug resistance and cancer progression in CRC (45, 46), but the specific mechanism of RNA polymerase II in chemoresistance should be explored in the future. The PI3K-Akt signaling and MAPK signaling pathways are important in the maintenance of cancer stem cells, which can mediate therapy resistance by dormancy, increased DNA repair, drug efflux, and so on (21). KRAS plays an important role in both the PI3K-Akt and MAPK signaling pathways and has been reported to promote chemoresistance in human cancers (25). Targeting KRAS could sensitize tumor cells to chemotherapy in CRC (47) and other cancers (48). The expression level of PTEN, a negative regulator of the PI3K signaling pathway, was also verified to be dysregulated in the chemoresistance of CRC, which is consistent with previously reported results (18). Overall, our KEGG pathway analysis indicates that the abnormal activities of these pathways during chemoresistance may be caused by our selected miRNAs, and targeting these miRNAs may also reverse chemoresistance in CRC.

Other studies have focused on circulating miRNAs as biomarkers for chemoresistance in CRC patients (18, 49). However, it seems that the results are not consistent. The study by Jin et al. began with the analysis of differentially expressed exosomal miRNAs secreted by drug-resistant CRC cell lines. All of the blood samples were collected before chemotherapy. The panel of exosomal miRNAs verified in their study was efficient in predicting the occurrence of chemoresistance. Our research started with the analysis of plasma exosomes in different CRC patients and, for the first time, demonstrated a panel of miRNAs that changes with the response to chemotherapy. Tumor cells need to undergo a series of changes to acquire the ability of resistance to chemotherapy (50, 51). The research by Jin et al. only differentiated patients with primary resistance. Our study focused on the miRNAs that change during chemotherapy and may be applied to a wider range of patients. Interestingly, they also revealed that the PI3K-Akt and MAPK signaling pathways may be crucial downstream regulatory pathways. Our experimental results were consistent with those of Jin et al.: PTEN is downregulated during chemoresistance. We also demonstrated that KRAS was positively related to chemoresistance in CRC cells and tissues. The regulation network of miRNAs is extremely intricate. The different origins of our specimens may be the reason why we identified different miRNAs, but the same targeted signaling pathway may reveal a crucial mechanism of chemoresistance in CRC.

Our research only studied CRC patients at our hospital, and the results should be tested in a multicenter study. Despite our results showing a relatively higher diagnostic efficiency, we only validated the miRNAs with the most significant changes (fold change >2 in upregulated miRNAs and <0.2 in downregulated miRNAs), which means that we may not have verified all of the dysregulated miRNAs in the chemoresistance of CRC patients. More results of the miRNA microarray analysis are shown in Supplementary Table 2. Whether combinations with other miRNAs could improve the diagnostic accuracy remains to be studied. Moreover, our study only focused on CRC patients receiving oxaliplatin-based chemotherapy (FOLFOX or XELOX). Whether our results are applicable to CRC patients undergoing other chemotherapies still needs further investigation.

In summary, we performed a well-designed and detailed analysis of the plasma exosomal miRNA profile in CRC patients receiving chemotherapy and identified changes in the levels of miR-100, miR-92a, miR-16, miR-30e, miR-144-5p, and let-7i between responsive and resistant patients. These results suggest that plasma exosomal miRNAs may serve as promising biomarkers for monitoring patients receiving chemotherapy.

## Data Availability Statement

The raw data supporting the conclusions of this article will be made available by the authors, without undue reservation, to any qualified researcher.

## Ethics Statement

The studies involving human participants were reviewed and approved by the Ethics Committee of Tianjin Medical University Cancer Institute and Hospital. The patients/participants provided their written informed consent to participate in this study. Written informed consent was obtained from the individual(s) for the publication of any potentially identifiable images or data included in this article.

## Author Contributions

YB and XC designed the study. RL and HZ collected the plasma samples as well as their clinical information. JH and WS carried out the experiments. JH and ZZ performed the data analysis. JH wrote the manuscript. All authors read and approved the final manuscript.

## Conflict of Interest

The authors declare that the research was conducted in the absence of any commercial or financial relationships that could be construed as a potential conflict of interest.
