# Supplementary material for: Plasma Exosomal miRNA Expression Profile as Oxaliplatin-Based Chemoresistant Biomarkers in Colorectal Adenocarcinoma
Source: Front Oncol. 2020 Sep 18;10:1495. doi: 10.3389/fonc.2020.01495 (PMC7531016; doi:10.3389/fonc.2020.01495)
Supplement: Supplementary file 1 [file Data_Sheet_1.docx]

Supplemental Data Methods

**Cell culture and tissue samples**

Colorectal cancer cell lines SW480 and HCT116 were obtained from Shanghai Institute of Cell Biology (Shanghai, China). We continuously exposed the parental cell lines to increasing concentrations of oxaliplatin for 10 months as described previously(1) and obtained the oxaliplatin-resistant cell lines SW480/LOHP and HCT116/LOHP. SW480 and SW480/LOHP cells were cultured in RPMI-1640 medium with 10% FBS (Gibco, Carlsbad, CA, USA). HCT116 and HCT116/LOHP cells were cultured in DMEM with 10% FBS. All cells were placed in a humidified atmosphere with 5% CO_2_ at 37℃ to a proper density for the next step.

All colorectal cancer tissues were obtained from Tianjin Medical University Cancer Institute and Hospital (Tianjin, China). Written consent was obtained from every patient and the Ethics Committee of Tianjin Medical University Cancer Institute and Hospital approved all aspects of this study.

**Quantification of miRNAs by probe-based RT-qPCR**

The TaqMan probe-based RT-qPCR assay was performed according to the manufacturer’s instructions (Applied Biosystems). The Ct values were determined by fixed threshold settings. The reverse transcription reaction was performed in a 10μL system containing 2μL extracted RNA, 1μL 10 mmol/L dNTPs, 0.5μL AMV reverse transcriptase (TaKaRa), 1μL stem-loop RT primer (Applied Biosystems), 2μL 5×reverse transcription buffer and 3.5μL DEPC water. The reverse transcription program was 16℃ for 30min, 42℃ for 30min, 85℃ for 5min and then held at 4℃. The real-time PCR was performed in a 20μL reaction system containing 2μL of cDNA, 0.3μL of Taq, 0.33μL of hydrolysis probe (Applied Biosystems), 1.2μL of 25 mmol/L MgCl_2_, 0.4μL of 10mmol/L dNTPs, 2μL of 10×PCR buffer and 13.77μL of DEPC water. Real-time PCR program was 1 cycle of 95℃ for 5min and 40 cycles of 95℃ for 15sec and 60℃ for 1min and was performed by LightCycler®96 (Roche). All results were analyzed in triplicate. To reduce the batch effect, the samples of the 3 groups were analyzed simultaneously on 96-well plates.

**Risk score analysis**

The risk score was defined as 1 if the candidate upregulated miRNA expression level was above the upper 95% reference interval in the response group or the candidate downregulated miRNA expression level was below the lower 5% reference interval in the response group. Otherwise, the risk score was defined as 0. We then established a risk score function (RSF) to predict chemoresistance in accordance with a linear combination of the expression level of each miRNA. For example, S_ij_ represents the risk score for miRNA j on sample I, and W_j_ denotes the weight of the risk score of miRNA j. The RSF for sample I using information from 6 miRNAs was calculated as:

$$\Sigma_{j-1}^{6}w_{j}s_{ij}$$

The W_j_ was calculated by a univariate logistic regression model based on the disease status generated by each of the risk scores of miRNA j. The regression coefficient for each risk score served as the weight to identify the contribution of

each miRNA to the RSF.

**Gene Ontology (GO) and Kyoto Encyclopedia of Genes and Genomes (KEGG) pathways**

The candidate miRNAs were uploaded to TargetScan (<http://www.targetscan.org/vert_72/>), miRDB (<http://mirdb.org/>) and microT-CDS (<http://diana.imis.athena-innovation.gr/>), and the common target genes were selected for further analysis. An online bioinformatics tool, DAVID Bioinformatics Resources 6.8 (<https://david.ncifcrf.gov/>), was used to perform GO analysis and KEGG pathway analysis. All visualizations were carried out by RStudio (version 1.2.5033). *P*<0.05 indicated significant enrichment between the compared groups.

1. Sun W, Li J, Zhou L, Han J, Liu R, Zhang H, et al. The c-Myc/miR-27b-3p/ATG10 regulatory axis regulates chemoresistance in colorectal cancer. *Theranostics* (2020) 10(5):1981-96. Epub 2020/02/28. doi: 10.7150/thno.37621. PubMed PMID: 32104496; PubMed Central PMCID: PMCPMC7019154.

Supplemental Table 1

The size distrigbution of exosomes, analyzed by NanoSight.

| Bin centre (nm) | Intensity (percent) | Bin centre (nm) | Intensity (percent) | Bin centre (nm) | Intensity (percent) | Bin centre (nm) | Intensity (percent) |
| --- | --- | --- | --- | --- | --- | --- | --- |
| 0.5-36.5 | 0 | 253.5 | 0.12084 | 470.5 | 0.0148 | 687.5 | 4.76E-05 |
| 37.5 | 8.1E-08 | 254.5 | 0.11956 | 471.5 | 0.015 | 688.5 | 5.08E-05 |
| 38.5 | 1.6E-07 | 255.5 | 0.11833 | 472.5 | 0.0152 | 689.5 | 5.42E-05 |
| 39.5 | 2.4E-07 | 256.5 | 0.11716 | 473.5 | 0.01541 | 690.5 | 5.79E-05 |
| 40.5 | 4.1E-07 | 257.5 | 0.11604 | 474.5 | 0.01561 | 691.5 | 6.19E-05 |
| 41.5 | 5.7E-07 | 258.5 | 0.11498 | 475.5 | 0.01582 | 692.5 | 6.62E-05 |
| 42.5 | 8.9E-07 | 259.5 | 0.11397 | 476.5 | 0.01603 | 693.5 | 7.09E-05 |
| 43.5 | 1.5E-06 | 260.5 | 0.11301 | 477.5 | 0.01624 | 694.5 | 7.59E-05 |
| 44.5 | 2.4E-06 | 261.5 | 0.1121 | 478.5 | 0.01646 | 695.5 | 8.13E-05 |
| 45.5 | 3.6E-06 | 262.5 | 0.11124 | 479.5 | 0.01667 | 696.5 | 8.72E-05 |
| 46.5 | 5.8E-06 | 263.5 | 0.11043 | 480.5 | 0.01689 | 697.5 | 9.35E-05 |
| 47.5 | 9.1E-06 | 264.5 | 0.10966 | 481.5 | 0.01711 | 698.5 | 0.0001 |
| 48.5 | 1.5E-05 | 265.5 | 0.10893 | 482.5 | 0.01733 | 699.5 | 0.000108 |
| 49.5 | 2.3E-05 | 266.5 | 0.10825 | 483.5 | 0.01754 | 700.5 | 0.000116 |
| 50.5 | 3.6E-05 | 267.5 | 0.1076 | 484.5 | 0.01776 | 701.5 | 0.000124 |
| 51.5 | 5.6E-05 | 268.5 | 0.10699 | 485.5 | 0.01798 | 702.5 | 0.000133 |
| 52.5 | 8.8E-05 | 269.5 | 0.10641 | 486.5 | 0.0182 | 703.5 | 0.000143 |
| 53.5 | 0.00014 | 270.5 | 0.10587 | 487.5 | 0.01842 | 704.5 | 0.000154 |
| 54.5 | 0.00021 | 271.5 | 0.10535 | 488.5 | 0.01863 | 705.5 | 0.000165 |
| 55.5 | 0.00033 | 272.5 | 0.10486 | 489.5 | 0.01885 | 706.5 | 0.000178 |
| 56.5 | 0.0005 | 273.5 | 0.10439 | 490.5 | 0.01906 | 707.5 | 0.000191 |
| 57.5 | 0.00077 | 274.5 | 0.10395 | 491.5 | 0.01927 | 708.5 | 0.000205 |
| 58.5 | 0.00117 | 275.5 | 0.10352 | 492.5 | 0.01948 | 709.5 | 0.00022 |
| 59.5 | 0.00177 | 276.5 | 0.10311 | 493.5 | 0.01968 | 710.5 | 0.000237 |
| 60.5 | 0.00264 | 277.5 | 0.10271 | 494.5 | 0.01989 | 711.5 | 0.000254 |
| 61.5 | 0.00392 | 278.5 | 0.10233 | 495.5 | 0.02009 | 712.5 | 0.000273 |
| 62.5 | 0.00575 | 279.5 | 0.10195 | 496.5 | 0.02028 | 713.5 | 0.000293 |
| 63.5 | 0.00834 | 280.5 | 0.10158 | 497.5 | 0.02047 | 714.5 | 0.000315 |
| 64.5 | 0.01195 | 281.5 | 0.1012 | 498.5 | 0.02065 | 715.5 | 0.000338 |
| 65.5 | 0.01692 | 282.5 | 0.10083 | 499.5 | 0.02083 | 716.5 | 0.000362 |
| 66.5 | 0.02363 | 283.5 | 0.10046 | 500.5 | 0.02101 | 717.5 | 0.000389 |
| 67.5 | 0.03254 | 284.5 | 0.10008 | 501.5 | 0.02118 | 718.5 | 0.000417 |
| 68.5 | 0.04416 | 285.5 | 0.09969 | 502.5 | 0.02134 | 719.5 | 0.000447 |
| 69.5 | 0.05903 | 286.5 | 0.09928 | 503.5 | 0.0215 | 720.5 | 0.000478 |
| 70.5 | 0.07767 | 287.5 | 0.09887 | 504.5 | 0.02164 | 721.5 | 0.000512 |
| 71.5 | 0.10057 | 288.5 | 0.09844 | 505.5 | 0.02179 | 722.5 | 0.000548 |
| 72.5 | 0.12814 | 289.5 | 0.09799 | 506.5 | 0.02192 | 723.5 | 0.000587 |
| 73.5 | 0.16059 | 290.5 | 0.09751 | 507.5 | 0.02205 | 724.5 | 0.000627 |
| 74.5 | 0.19797 | 291.5 | 0.09702 | 508.5 | 0.02216 | 725.5 | 0.00067 |
| 75.5 | 0.24005 | 292.5 | 0.09649 | 509.5 | 0.02227 | 726.5 | 0.000716 |
| 76.5 | 0.33633 | 293.5 | 0.09594 | 510.5 | 0.01471 | 727.5 | 0.000764 |
| 77.5 | 0.38604 | 294.5 | 0.09536 | 511.5 | 0.01453 | 728.5 | 0.000815 |
| 78.5 | 0.43812 | 295.5 | 0.09475 | 512.5 | 0.01434 | 729.5 | 0.000868 |
| 79.5 | 0.49132 | 296.5 | 0.09411 | 513.5 | 0.01413 | 730.5 | 0.000925 |
| 80.5 | 0.54424 | 297.5 | 0.09343 | 514.5 | 0.01392 | 731.5 | 0.000985 |
| 81.5 | 0.59543 | 298.5 | 0.09271 | 515.5 | 0.0137 | 732.5 | 0.001047 |
| 82.5 | 0.64347 | 299.5 | 0.09196 | 516.5 | 0.01346 | 733.5 | 0.001113 |
| 83.5 | 0.6871 | 300.5 | 0.09117 | 517.5 | 0.01322 | 734.5 | 0.001182 |
| 84.5 | 0.72528 | 301.5 | 0.09034 | 518.5 | 0.01297 | 735.5 | 0.001254 |
| 85.5 | 0.75722 | 302.5 | 0.08948 | 519.5 | 0.01271 | 736.5 | 0.00133 |
| 86.5 | 0.78248 | 303.5 | 0.08857 | 520.5 | 0.01244 | 737.5 | 0.001409 |
| 87.5 | 0.80092 | 304.5 | 0.08762 | 521.5 | 0.01216 | 738.5 | 0.001491 |
| 88.5 | 0.81271 | 305.5 | 0.08663 | 522.5 | 0.01188 | 739.5 | 0.001577 |
| 89.5 | 0.81828 | 306.5 | 0.08561 | 523.5 | 0.01159 | 740.5 | 0.001666 |
| 90.5 | 0.81827 | 307.5 | 0.08454 | 524.5 | 0.0113 | 741.5 | 0.001759 |
| 91.5 | 0.81345 | 308.5 | 0.08344 | 525.5 | 0.011 | 742.5 | 0.001855 |
| 92.5 | 0.83327 | 309.5 | 0.0823 | 526.5 | 0.01069 | 743.5 | 0.001954 |
| 93.5 | 0.85835 | 310.5 | 0.08113 | 527.5 | 0.01039 | 744.5 | 0.002057 |
| 94.5 | 0.88412 | 311.5 | 0.07992 | 528.5 | 0.01008 | 745.5 | 0.002164 |
| 95.5 | 0.91034 | 312.5 | 0.07868 | 529.5 | 0.00977 | 746.5 | 0.002273 |
| 96.5 | 0.93672 | 313.5 | 0.0774 | 530.5 | 0.00945 | 747.5 | 0.002386 |
| 97.5 | 0.96301 | 314.5 | 0.0761 | 531.5 | 0.00914 | 748.5 | 0.002501 |
| 98.5 | 0.98892 | 315.5 | 0.07477 | 532.5 | 0.00883 | 749.5 | 0.00262 |
| 99.5 | 1.01421 | 316.5 | 0.07341 | 533.5 | 0.00851 | 750.5 | 0.002741 |
| 100.5 | 1.03863 | 317.5 | 0.07204 | 534.5 | 0.0082 | 751.5 | 0.002865 |
| 101.5 | 1.06195 | 318.5 | 0.07064 | 535.5 | 0.00789 | 752.5 | 0.002991 |
| 102.5 | 1.08398 | 319.5 | 0.06922 | 536.5 | 0.00759 | 753.5 | 0.00312 |
| 103.5 | 1.10454 | 320.5 | 0.06779 | 537.5 | 0.00729 | 754.5 | 0.00325 |
| 104.5 | 1.12348 | 321.5 | 0.06634 | 538.5 | 0.00699 | 755.5 | 0.003382 |
| 105.5 | 1.1407 | 322.5 | 0.06488 | 539.5 | 0.00669 | 756.5 | 0.003516 |
| 106.5 | 1.15611 | 323.5 | 0.06342 | 540.5 | 0.0064 | 757.5 | 0.003651 |
| 107.5 | 1.16965 | 324.5 | 0.06195 | 541.5 | 0.00612 | 758.5 | 0.003786 |
| 108.5 | 1.18129 | 325.5 | 0.06048 | 542.5 | 0.00584 | 759.5 | 0.003922 |
| 109.5 | 1.19102 | 326.5 | 0.05901 | 543.5 | 0.00557 | 760.5 | 0.004059 |
| 110.5 | 1.19885 | 327.5 | 0.05754 | 544.5 | 0.0053 | 761.5 | 0.004195 |
| 111.5 | 1.20481 | 328.5 | 0.05607 | 545.5 | 0.00504 | 762.5 | 0.004331 |
| 112.5 | 1.20893 | 329.5 | 0.05461 | 546.5 | 0.00479 | 763.5 | 0.004466 |
| 113.5 | 1.21127 | 330.5 | 0.05317 | 547.5 | 0.00455 | 764.5 | 0.0046 |
| 114.5 | 1.21186 | 331.5 | 0.05173 | 548.5 | 0.00431 | 765.5 | 0.004732 |
| 115.5 | 1.21077 | 332.5 | 0.05031 | 549.5 | 0.00408 | 766.5 | 0.004862 |
| 116.5 | 1.20804 | 333.5 | 0.0489 | 550.5 | 0.00386 | 767.5 | 0.004989 |
| 117.5 | 1.20371 | 334.5 | 0.04752 | 551.5 | 0.00365 | 768.5 | 0.005114 |
| 118.5 | 1.19784 | 335.5 | 0.04615 | 552.5 | 0.00344 | 769.5 | 0.005236 |
| 119.5 | 1.19046 | 336.5 | 0.0448 | 553.5 | 0.00324 | 770.5 | 0.005353 |
| 120.5 | 1.1816 | 337.5 | 0.04348 | 554.5 | 0.00305 | 771.5 | 0.005467 |
| 121.5 | 1.1713 | 338.5 | 0.04218 | 555.5 | 0.00287 | 772.5 | 0.005577 |
| 122.5 | 1.15958 | 339.5 | 0.04091 | 556.5 | 0.0027 | 773.5 | 0.005681 |
| 123.5 | 1.14647 | 340.5 | 0.03966 | 557.5 | 0.00254 | 774.5 | 0.00578 |
| 124.5 | 1.13199 | 341.5 | 0.03845 | 558.5 | 0.00238 | 775.5 | 0.005874 |
| 125.5 | 1.11618 | 342.5 | 0.03726 | 559.5 | 0.00223 | 776.5 | 0.005962 |
| 126.5 | 1.09905 | 343.5 | 0.0361 | 560.5 | 0.00209 | 777.5 | 0.006043 |
| 127.5 | 1.08066 | 344.5 | 0.03497 | 561.5 | 0.00195 | 778.5 | 0.006118 |
| 128.5 | 1.06103 | 345.5 | 0.03387 | 562.5 | 0.00182 | 779.5 | 0.006186 |
| 129.5 | 1.04022 | 346.5 | 0.0328 | 563.5 | 0.0017 | 780.5 | 0.006247 |
| 130.5 | 1.01828 | 347.5 | 0.03176 | 564.5 | 0.00159 | 781.5 | 0.0063 |
| 131.5 | 0.99529 | 348.5 | 0.03075 | 565.5 | 0.00148 | 782.5 | 0.006346 |
| 132.5 | 0.97133 | 349.5 | 0.02978 | 566.5 | 0.00138 | 783.5 | 0.006384 |
| 133.5 | 0.94649 | 350.5 | 0.02883 | 567.5 | 0.00128 | 784.5 | 0.006413 |
| 134.5 | 0.92085 | 351.5 | 0.02792 | 568.5 | 0.00119 | 785.5 | 0.006435 |
| 135.5 | 0.89455 | 352.5 | 0.02704 | 569.5 | 0.00111 | 786.5 | 0.006449 |
| 136.5 | 0.86768 | 353.5 | 0.02619 | 570.5 | 0.00103 | 787.5 | 0.006454 |
| 137.5 | 0.84037 | 354.5 | 0.02536 | 571.5 | 0.00095 | 788.5 | 0.006451 |
| 138.5 | 0.81275 | 355.5 | 0.02457 | 572.5 | 0.00088 | 789.5 | 0.00644 |
| 139.5 | 0.78495 | 356.5 | 0.02381 | 573.5 | 0.00082 | 790.5 | 0.006421 |
| 140.5 | 0.7571 | 357.5 | 0.02308 | 574.5 | 0.00076 | 791.5 | 0.006393 |
| 141.5 | 0.72933 | 358.5 | 0.02237 | 575.5 | 0.0007 | 792.5 | 0.006357 |
| 142.5 | 0.70176 | 359.5 | 0.02169 | 576.5 | 0.00065 | 793.5 | 0.006313 |
| 143.5 | 0.65451 | 360.5 | 0.02104 | 577.5 | 0.0006 | 794.5 | 0.006262 |
| 144.5 | 0.6477 | 361.5 | 0.02042 | 578.5 | 0.00056 | 795.5 | 0.006203 |
| 145.5 | 0.62143 | 362.5 | 0.01982 | 579.5 | 0.00051 | 796.5 | 0.006136 |
| 146.5 | 0.59579 | 363.5 | 0.01924 | 580.5 | 0.00047 | 797.5 | 0.006062 |
| 147.5 | 0.57087 | 364.5 | 0.01869 | 581.5 | 0.00044 | 798.5 | 0.005982 |
| 148.5 | 0.54675 | 365.5 | 0.01817 | 582.5 | 0.0004 | 799.5 | 0.005895 |
| 149.5 | 0.50349 | 366.5 | 0.01766 | 583.5 | 0.00037 | 800.5 | 0.005801 |
| 150.5 | 0.45113 | 367.5 | 0.01718 | 584.5 | 0.00034 | 801.5 | 0.005702 |
| 151.5 | 0.45971 | 368.5 | 0.01672 | 585.5 | 0.00032 | 802.5 | 0.005597 |
| 152.5 | 0.43928 | 369.5 | 0.01628 | 586.5 | 0.00029 | 803.5 | 0.005487 |
| 153.5 | 0.38983 | 370.5 | 0.01586 | 587.5 | 0.00027 | 804.5 | 0.005372 |
| 154.5 | 0.37139 | 371.5 | 0.01546 | 588.5 | 0.00025 | 805.5 | 0.005253 |
| 155.5 | 0.35395 | 372.5 | 0.01508 | 589.5 | 0.00023 | 806.5 | 0.005129 |
| 156.5 | 0.33751 | 373.5 | 0.01471 | 590.5 | 0.00021 | 807.5 | 0.005002 |
| 157.5 | 0.32205 | 374.5 | 0.01436 | 591.5 | 0.0002 | 808.5 | 0.004872 |
| 158.5 | 0.30756 | 375.5 | 0.01403 | 592.5 | 0.00018 | 809.5 | 0.00474 |
| 159.5 | 0.294 | 376.5 | 0.01372 | 593.5 | 0.00017 | 810.5 | 0.004604 |
| 160.5 | 0.28136 | 377.5 | 0.01342 | 594.5 | 0.00015 | 811.5 | 0.004467 |
| 161.5 | 0.26959 | 378.5 | 0.01314 | 595.5 | 0.00014 | 812.5 | 0.004329 |
| 162.5 | 0.25868 | 379.5 | 0.01287 | 596.5 | 0.00013 | 813.5 | 0.004189 |
| 163.5 | 0.24858 | 380.5 | 0.01261 | 597.5 | 0.00012 | 814.5 | 0.004049 |
| 164.5 | 0.23925 | 381.5 | 0.01237 | 598.5 | 0.00011 | 815.5 | 0.003908 |
| 165.5 | 0.23067 | 382.5 | 0.01214 | 599.5 | 0.0001 | 816.5 | 0.003767 |
| 166.5 | 0.22279 | 383.5 | 0.01192 | 600.5 | 9.6E-05 | 817.5 | 0.003627 |
| 167.5 | 0.21559 | 384.5 | 0.01171 | 601.5 | 8.9E-05 | 818.5 | 0.003487 |
| 168.5 | 0.20901 | 385.5 | 0.01151 | 602.5 | 8.2E-05 | 819.5 | 0.003349 |
| 169.5 | 0.20304 | 386.5 | 0.01133 | 603.5 | 7.6E-05 | 820.5 | 0.003212 |
| 170.5 | 0.19764 | 387.5 | 0.01116 | 604.5 | 7.1E-05 | 821.5 | 0.003077 |
| 171.5 | 0.19277 | 388.5 | 0.01099 | 605.5 | 6.6E-05 | 822.5 | 0.002943 |
| 172.5 | 0.18841 | 389.5 | 0.01084 | 606.5 | 6.1E-05 | 823.5 | 0.002812 |
| 173.5 | 0.18453 | 390.5 | 0.01069 | 607.5 | 5.7E-05 | 824.5 | 0.002683 |
| 174.5 | 0.1811 | 391.5 | 0.01055 | 608.5 | 5.3E-05 | 825.5 | 0.002557 |
| 175.5 | 0.17809 | 392.5 | 0.01042 | 609.5 | 4.9E-05 | 826.5 | 0.002434 |
| 176.5 | 0.17549 | 393.5 | 0.0103 | 610.5 | 4.6E-05 | 827.5 | 0.002313 |
| 177.5 | 0.17325 | 394.5 | 0.01019 | 611.5 | 4.3E-05 | 828.5 | 0.002196 |
| 178.5 | 0.17137 | 395.5 | 0.01008 | 612.5 | 4E-05 | 829.5 | 0.002082 |
| 179.5 | 0.16982 | 396.5 | 0.00999 | 613.5 | 3.8E-05 | 830.5 | 0.001972 |
| 180.5 | 0.16858 | 397.5 | 0.0099 | 614.5 | 3.5E-05 | 831.5 | 0.001865 |
| 181.5 | 0.16764 | 398.5 | 0.00981 | 615.5 | 3.3E-05 | 832.5 | 0.001762 |
| 182.5 | 0.16696 | 399.5 | 0.00973 | 616.5 | 3.1E-05 | 833.5 | 0.001662 |
| 183.5 | 0.16653 | 400.5 | 0.00966 | 617.5 | 2.9E-05 | 834.5 | 0.001566 |
| 184.5 | 0.16634 | 401.5 | 0.0096 | 618.5 | 2.8E-05 | 835.5 | 0.001474 |
| 185.5 | 0.16636 | 402.5 | 0.00954 | 619.5 | 2.6E-05 | 836.5 | 0.001385 |
| 186.5 | 0.16658 | 403.5 | 0.00948 | 620.5 | 2.5E-05 | 837.5 | 0.0013 |
| 187.5 | 0.16698 | 404.5 | 0.00943 | 621.5 | 2.3E-05 | 838.5 | 0.001219 |
| 188.5 | 0.16754 | 405.5 | 0.00939 | 622.5 | 2.2E-05 | 839.5 | 0.001142 |
| 189.5 | 0.16825 | 406.5 | 0.00935 | 623.5 | 2.1E-05 | 840.5 | 0.001068 |
| 190.5 | 0.16909 | 407.5 | 0.00932 | 624.5 | 2E-05 | 841.5 | 0.000997 |
| 191.5 | 0.17003 | 408.5 | 0.00929 | 625.5 | 1.9E-05 | 842.5 | 0.00093 |
| 192.5 | 0.17107 | 409.5 | 0.00927 | 626.5 | 1.8E-05 | 843.5 | 0.000867 |
| 193.5 | 0.17219 | 410.5 | 0.00925 | 627.5 | 1.8E-05 | 844.5 | 0.000807 |
| 194.5 | 0.17336 | 411.5 | 0.00923 | 628.5 | 1.7E-05 | 845.5 | 0.00075 |
| 195.5 | 0.17458 | 412.5 | 0.00922 | 629.5 | 1.6E-05 | 846.5 | 0.000696 |
| 196.5 | 0.17582 | 413.5 | 0.00922 | 630.5 | 1.6E-05 | 847.5 | 0.000646 |
| 197.5 | 0.17707 | 414.5 | 0.00922 | 631.5 | 1.5E-05 | 848.5 | 0.000598 |
| 198.5 | 0.17831 | 415.5 | 0.00922 | 632.5 | 1.5E-05 | 849.5 | 0.000553 |
| 199.5 | 0.17953 | 416.5 | 0.00922 | 633.5 | 1.4E-05 | 850.5 | 0.000511 |
| 200.5 | 0.18071 | 417.5 | 0.00923 | 634.5 | 1.4E-05 | 851.5 | 0.000472 |
| 201.5 | 0.18184 | 418.5 | 0.00925 | 635.5 | 1.3E-05 | 852.5 | 0.000435 |
| 202.5 | 0.18289 | 419.5 | 0.00927 | 636.5 | 1.3E-05 | 853.5 | 0.0004 |
| 203.5 | 0.18387 | 420.5 | 0.00929 | 637.5 | 1.3E-05 | 854.5 | 0.000368 |
| 204.5 | 0.18475 | 421.5 | 0.00931 | 638.5 | 1.2E-05 | 855.5 | 0.000338 |
| 205.5 | 0.18553 | 422.5 | 0.00934 | 639.5 | 1.2E-05 | 856.5 | 0.00031 |
| 206.5 | 0.1862 | 423.5 | 0.00937 | 640.5 | 1.2E-05 | 857.5 | 0.000284 |
| 207.5 | 0.18674 | 424.5 | 0.00941 | 641.5 | 1.2E-05 | 858.5 | 0.00026 |
| 208.5 | 0.18715 | 425.5 | 0.00945 | 642.5 | 1.2E-05 | 859.5 | 0.000237 |
| 209.5 | 0.18743 | 426.5 | 0.00949 | 643.5 | 1.2E-05 | 860.5 | 0.000217 |
| 210.5 | 0.18756 | 427.5 | 0.00953 | 644.5 | 1.1E-05 | 861.5 | 0.000198 |
| 211.5 | 0.18755 | 428.5 | 0.00958 | 645.5 | 1.1E-05 | 862.5 | 0.00018 |
| 212.5 | 0.18739 | 429.5 | 0.00964 | 646.5 | 1.1E-05 | 863.5 | 0.000164 |
| 213.5 | 0.18709 | 430.5 | 0.00969 | 647.5 | 1.1E-05 | 864.5 | 0.000149 |
| 214.5 | 0.18663 | 431.5 | 0.00975 | 648.5 | 1.1E-05 | 865.5 | 0.000135 |
| 215.5 | 0.18603 | 432.5 | 0.00981 | 649.5 | 1.1E-05 | 866.5 | 0.000122 |
| 216.5 | 0.18529 | 433.5 | 0.00988 | 650.5 | 1.1E-05 | 867.5 | 0.000111 |
| 217.5 | 0.1844 | 434.5 | 0.00995 | 651.5 | 1.1E-05 | 868.5 | 0.0001 |
| 218.5 | 0.18338 | 435.5 | 0.01002 | 652.5 | 1.2E-05 | 869.5 | 9.06E-05 |
| 219.5 | 0.18223 | 436.5 | 0.0101 | 653.5 | 1.2E-05 | 870.5 | 8.17E-05 |
| 220.5 | 0.18096 | 437.5 | 0.01018 | 654.5 | 1.2E-05 | 871.5 | 7.36E-05 |
| 221.5 | 0.17957 | 438.5 | 0.01026 | 655.5 | 1.2E-05 | 872.5 | 6.64E-05 |
| 222.5 | 0.17808 | 439.5 | 0.01035 | 656.5 | 1.2E-05 | 873.5 | 5.96E-05 |
| 223.5 | 0.17648 | 440.5 | 0.01044 | 657.5 | 1.2E-05 | 874.5 | 5.36E-05 |
| 224.5 | 0.1748 | 441.5 | 0.01053 | 658.5 | 1.3E-05 | 875.5 | 4.81E-05 |
| 225.5 | 0.17303 | 442.5 | 0.01063 | 659.5 | 1.3E-05 | 876.5 | 4.31E-05 |
| 226.5 | 0.17119 | 443.5 | 0.01073 | 660.5 | 1.3E-05 | 877.5 | 3.86E-05 |
| 227.5 | 0.16928 | 444.5 | 0.01083 | 661.5 | 1.4E-05 | 878.5 | 3.45E-05 |
| 228.5 | 0.16732 | 445.5 | 0.01094 | 662.5 | 1.4E-05 | 879.5 | 3.08E-05 |
| 229.5 | 0.16532 | 446.5 | 0.01105 | 663.5 | 1.4E-05 | 880.5 | 2.75E-05 |
| 230.5 | 0.16327 | 447.5 | 0.01117 | 664.5 | 1.5E-05 | 881.5 | 2.45E-05 |
| 231.5 | 0.1612 | 448.5 | 0.01128 | 665.5 | 1.5E-05 | 882.5 | 2.18E-05 |
| 232.5 | 0.15911 | 449.5 | 0.01141 | 666.5 | 1.6E-05 | 883.5 | 1.94E-05 |
| 233.5 | 0.15701 | 450.5 | 0.01153 | 667.5 | 1.7E-05 | 884.5 | 1.72E-05 |
| 234.5 | 0.1549 | 451.5 | 0.01166 | 668.5 | 1.7E-05 | 885.5 | 1.53E-05 |
| 235.5 | 0.1528 | 452.5 | 0.01179 | 669.5 | 1.8E-05 | 886.5 | 1.35E-05 |
| 236.5 | 0.15071 | 453.5 | 0.01193 | 670.5 | 1.9E-05 | 887.5 | 0.000012 |
| 237.5 | 0.14863 | 454.5 | 0.01207 | 671.5 | 2E-05 | 888.5 | 1.06E-05 |
| 238.5 | 0.14657 | 455.5 | 0.01221 | 672.5 | 2.1E-05 | 889.5 | 9.39E-06 |
| 239.5 | 0.14454 | 456.5 | 0.01236 | 673.5 | 2.2E-05 | 890.5 | 8.25E-06 |
| 240.5 | 0.14254 | 457.5 | 0.01251 | 674.5 | 2.3E-05 | 891.5 | 7.28E-06 |
| 241.5 | 0.14058 | 458.5 | 0.01267 | 675.5 | 2.4E-05 | 892.5 | 6.39E-06 |
| 242.5 | 0.13865 | 459.5 | 0.01283 | 676.5 | 2.5E-05 | 893.5 | 5.66E-06 |
| 243.5 | 0.13677 | 460.5 | 0.01299 | 677.5 | 2.6E-05 | 894.5 | 4.94E-06 |
| 244.5 | 0.13494 | 461.5 | 0.01315 | 678.5 | 2.8E-05 | 895.5 | 4.37E-06 |
| 245.5 | 0.13315 | 462.5 | 0.01332 | 679.5 | 2.9E-05 | 896.5 | 3.8E-06 |
| 246.5 | 0.13142 | 463.5 | 0.0135 | 680.5 | 3.1E-05 | 897.5 | 3.32E-06 |
| 247.5 | 0.12974 | 464.5 | 0.01367 | 681.5 | 3.3E-05 | 898.5 | 2.91E-06 |
| 248.5 | 0.12812 | 465.5 | 0.01385 | 682.5 | 3.5E-05 | 899.5 | 2.59E-06 |
| 249.5 | 0.12655 | 466.5 | 0.01404 | 683.5 | 3.7E-05 | 900-1000 | 0 |
| 250.5 | 0.12503 | 467.5 | 0.01422 | 684.5 | 3.9E-05 |  |  |
| 251.5 | 0.12358 | 468.5 | 0.01441 | 685.5 | 4.2E-05 |  |  |
| 252.5 | 0.12218 | 469.5 | 0.01461 | 686.5 | 4.5E-05 |  |  |

Supplemental Table 2

Significantly dysregulated miRNAs in plasma exosomes of resistant patients compared to responsive patients by miRNA microarray analysis.

| **miRNA** | **Resistance** | **Response** | **Fold Change (Rs/Rp)** |
| --- | --- | --- | --- |
| hsa-miR-184 | 4796 | 5 | 959.20 |
| hsa-miR-100-5p | 1867 | 180 | 10.37 |
| hsa-miR-10a-5p | 13279 | 4549 | 2.92 |
| hsa-miR-92a-3p | 19383 | 7775 | 2.49 |
| hsa-miR-15b-5p | 256 | 104 | 2.46 |
| hsa-miR-24-3p | 631 | 296 | 2.13 |
| hsa-miR-23a-3p | 648 | 337 | 1.92 |
| hsa-miR-423-3p | 984 | 524 | 1.88 |
| hsa-miR-150-5p | 5301 | 2867 | 1.85 |
| hsa-miR-30e-3p | 183 | 113 | 1.62 |
| hsa-let-7d-3p | 208 | 129 | 1.61 |
| hsa-miR-128-3p | 282 | 203 | 1.39 |
| hsa-let-7b-5p | 1379 | 1012 | 1.36 |
| hsa-miR-146a-5p | 1353 | 1000 | 1.35 |
| hsa-miR-484 | 570 | 428 | 1.33 |
| hsa-miR-425-5p | 454 | 363 | 1.25 |
| hsa-miR-139-5p | 132 | 111 | 1.19 |
| hsa-miR-99b-5p | 447 | 389 | 1.15 |
| hsa-miR-92b-3p | 237 | 208 | 1.14 |
| hsa-miR-25-3p | 3329 | 2967 | 1.12 |
| hsa-miR-15a-5p | 1594 | 1423 | 1.12 |
| hsa-miR-221-3p | 626 | 596 | 1.05 |
| hsa-miR-223-3p | 893 | 856 | 1.04 |
| hsa-miR-222-3p | 409 | 396 | 1.03 |
| hsa-miR-151a-5p | 550 | 537 | 1.02 |
| hsa-miR-125a-5p | 1555 | 1547 | 1.01 |
| hsa-miR-28-3p | 624 | 711 | 0.88 |
| hsa-miR-30a-3p | 117 | 140 | 0.84 |
| hsa-miR-423-5p | 1265 | 1518 | 0.83 |
| hsa-miR-140-3p | 864 | 1039 | 0.83 |
| hsa-miR-151a-3p | 856 | 1085 | 0.79 |
| hsa-miR-342-3p | 240 | 309 | 0.78 |
| hsa-miR-182-5p | 999 | 1288 | 0.78 |
| hsa-miR-125b-5p | 403 | 541 | 0.74 |
| hsa-miR-199a-3p | 618 | 874 | 0.71 |
| hsa-miR-27b-3p | 531 | 760 | 0.70 |
| hsa-miR-107 | 242 | 357 | 0.68 |
| hsa-let-7a-5p | 1377 | 2091 | 0.66 |
| hsa-miR-148b-3p | 218 | 341 | 0.64 |
| hsa-miR-106b-3p | 312 | 525 | 0.59 |
| hsa-miR-146b-5p | 239 | 414 | 0.58 |
| hsa-miR-451a | 7882 | 14168 | 0.56 |
| hsa-miR-30c-5p | 729 | 1311 | 0.56 |
| hsa-miR-30d-5p | 1419 | 2569 | 0.55 |
| hsa-miR-10b-5p | 5267 | 10018 | 0.53 |
| hsa-miR-148a-3p | 833 | 1593 | 0.52 |
| hsa-miR-486-3p | 257 | 498 | 0.52 |
| hsa-miR-30b-5p | 168 | 328 | 0.51 |
| hsa-let-7d-5p | 108 | 217 | 0.50 |
| hsa-miR-22-3p | 4342 | 8954 | 0.48 |
| hsa-miR-26a-5p | 4663 | 9764 | 0.48 |
| hsa-miR-103a-3p | 691 | 1483 | 0.47 |
| hsa-miR-27a-3p | 225 | 497 | 0.45 |
| hsa-miR-16-5p | 6836 | 15152 | 0.45 |
| hsa-miR-181b-5p | 122 | 297 | 0.41 |
| hsa-miR-29a-3p | 174 | 427 | 0.41 |
| hsa-miR-9-5p | 178 | 460 | 0.39 |
| hsa-miR-16-2-3p | 132 | 356 | 0.37 |
| hsa-miR-21-5p | 1347 | 3868 | 0.35 |
| hsa-miR-26b-5p | 736 | 2177 | 0.34 |
| hsa-let-7g-5p | 530 | 1737 | 0.31 |
| hsa-miR-181a-5p | 1015 | 3731 | 0.27 |
| hsa-miR-143-3p | 375 | 1398 | 0.27 |
| hsa-miR-378a-3p | 148 | 557 | 0.26 |
| hsa-miR-93-5p | 236 | 1064 | 0.22 |
| hsa-miR-142-5p | 1793 | 8102 | 0.22 |
| hsa-miR-126-3p | 1587 | 7336 | 0.22 |
| hsa-miR-30a-5p | 505 | 2521 | 0.20 |
| hsa-miR-192-5p | 143 | 723 | 0.20 |
| hsa-miR-101-3p | 372 | 1957 | 0.19 |
| hsa-let-7f-5p | 904 | 5038 | 0.18 |
| hsa-miR-144-3p | 394 | 2521 | 0.16 |
| hsa-miR-30e-5p | 509 | 3656 | 0.14 |
| hsa-miR-144-5p | 277 | 2063 | 0.13 |
| hsa-let-7i-5p | 275 | 2458 | 0.11 |
| hsa-miR-4532 | 86 | 50542 | 0.002 |

Rs: Resistance, Rp: Response.

Supplemental Table 3

Significantly upregulated miRNAs in plasma exosomes of resistant patients compared to responsive patients by miRNA microarray analysis.

| miRNA | Resistance | Response | Fold Change (Rs/Rp) |
| --- | --- | --- | --- |
| hsa-miR-184 | 4796 | 5 | 959.20 |
| hsa-miR-100-5p | 1867 | 180 | 10.37 |
| hsa-miR-10a-5p | 13278 | 4548 | 2.92 |
| hsa-miR-92a-3p | 19382 | 7775 | 2.49 |

Rs: Resistance, Rp: Response.

Supplemental Table 4

Significantly downregulated miRNAs in plasma exosomes of resistant patients compared to responsive patients by miRNA microarray analysis.

| miRNA | Resistance | Response | Fold Change (Rp/Rs) | |
| --- | --- | --- | --- | --- |
| hsa-miR-4532 | 86 | 50542 | 587.70 |  |
| hsa-let-7i-5p | 275 | 2458 | 8.94 | |
| hsa-miR-144-5p | 277 | 2063 | 7.45 | |
| hsa-miR-30e-5p | 509 | 3655 | 7.18 | |
| hsa-miR-144-3p | 394 | 2521 | 6.40 | |
| hsa-let-7f-5p | 904 | 5038 | 5.57 | |
| hsa-miR-101-3p | 372 | 1957 | 5.26 | |
| hsa-miR-30a-5p | 505 | 2520 | 4.99 | |
| hsa-miR-126-3p | 1587 | 7336 | 4.62 | |
| hsa-miR-142-5p | 1793 | 8102 | 4.52 | |
| hsa-miR-93-5p | 236 | 1064 | 4.51 | |
| hsa-miR-143-3p | 375 | 1398 | 3.73 | |
| hsa-miR-181a-5p | 1015 | 3731 | 3.68 | |
| hsa-let-7g-5p | 530 | 1737 | 3.28 | |
| hsa-miR-26b-5p | 736 | 2177 | 2.96 | |
| hsa-miR-21-5p | 1347 | 3868 | 2.87 | |
| hsa-miR-16-5p | 6836 | 15152 | 2.22 | |
| hsa-miR-103a-3p | 691 | 1482.5 | 2.15 | |
| hsa-miR-26a-5p | 4663 | 9764 | 2.09 | |
| hsa-miR-22-3p | 4342 | 8954 | 2.06 | |

Rs: Resistance, Rp: Response.

Supplemental Table 5

Quantification of RNA extracted from 210 blood samples by NanoDrop 2000 (Thermo, MA, USA)

| No. | Patients | A260/A280 | Conc.  (ng/μL) | No. | Patients | A260/A280 | Conc.  (ng/μL) |
| --- | --- | --- | --- | --- | --- | --- | --- |
| 1 | Control CRC | 1.95 | 48.0 | 106 | Response | 1.87 | 37.9 |
| 2 | Control CRC | 1.86 | 36.8 | 107 | Response | 1.92 | 29.5 |
| 3 | Control CRC | 2.09 | 23.0 | 108 | Response | 2.10 | 43.8 |
| 4 | Control CRC | 1.81 | 28.5 | 109 | Response | 2.08 | 36.7 |
| 5 | Control CRC | 1.95 | 37.0 | 110 | Response | 1.91 | 28.4 |
| 6 | Control CRC | 1.87 | 34.3 | 111 | Response | 1.98 | 42.5 |
| 7 | Control CRC | 1.80 | 39.6 | 112 | Response | 1.98 | 38.6 |
| 8 | Control CRC | 1.87 | 45.5 | 113 | Response | 1.93 | 30.0 |
| 9 | Control CRC | 2.08 | 46.4 | 114 | Response | 2.00 | 35.4 |
| 10 | Control CRC | 1.83 | 39.5 | 115 | Response | 2.05 | 31.8 |
| 11 | Control CRC | 2.08 | 38.2 | 116 | Response | 1.90 | 20.7 |
| 12 | Control CRC | 2.08 | 29.8 | 117 | Response | 1.87 | 29.5 |
| 13 | Control CRC | 1.82 | 20.7 | 118 | Response | 1.86 | 46.2 |
| 14 | Control CRC | 1.82 | 48.0 | 119 | Response | 1.88 | 22.2 |
| 15 | Control CRC | 1.82 | 52.2 | 120 | Response | 1.94 | 27.6 |
| 16 | Control CRC | 2.07 | 42.8 | 121 | Response | 1.81 | 49.6 |
| 17 | Control CRC | 1.99 | 42.4 | 122 | Response | 2.00 | 37.4 |
| 18 | Control CRC | 2.10 | 20.5 | 123 | Response | 2.06 | 30.4 |
| 19 | Control CRC | 2.09 | 19.4 | 124 | Response | 2.07 | 44.0 |
| 20 | Control CRC | 1.83 | 33.5 | 125 | Response | 2.09 | 32.2 |
| 21 | Control CRC | 1.82 | 50.6 | 126 | Response | 1.91 | 51.8 |
| 22 | Control CRC | 1.92 | 19.4 | 127 | Resistance | 2.04 | 29.3 |
| 23 | Control CRC | 2.05 | 37.9 | 128 | Resistance | 1.83 | 25.1 |
| 24 | Control CRC | 2.00 | 43.6 | 129 | Resistance | 2.10 | 19.8 |
| 25 | Control CRC | 1.98 | 37.6 | 130 | Resistance | 1.95 | 29.3 |
| 26 | Control CRC | 1.89 | 22.6 | 131 | Resistance | 1.88 | 19.3 |
| 27 | Control CRC | 1.89 | 30.5 | 132 | Resistance | 2.09 | 19.9 |
| 28 | Control CRC | 1.82 | 24.4 | 133 | Resistance | 1.87 | 45.3 |
| 29 | Control CRC | 1.98 | 48.6 | 134 | Resistance | 2.04 | 35.9 |
| 30 | Control CRC | 1.90 | 36.0 | 135 | Resistance | 1.81 | 38.2 |
| 31 | Control CRC | 1.89 | 24.4 | 136 | Resistance | 1.94 | 33.1 |
| 32 | Control CRC | 2.02 | 26.4 | 137 | Resistance | 1.99 | 50.4 |
| 33 | Control CRC | 1.85 | 19.7 | 138 | Resistance | 2.07 | 48.8 |
| 34 | Control CRC | 1.92 | 47.4 | 139 | Resistance | 2.07 | 22.5 |
| 35 | Control CRC | 1.86 | 21.8 | 140 | Resistance | 1.83 | 20.1 |
| 36 | Control CRC | 1.91 | 48.6 | 141 | Resistance | 1.96 | 45.1 |
| 37 | Control CRC | 1.93 | 41.1 | 142 | Resistance | 1.84 | 25.0 |
| 38 | Control CRC | 2.01 | 51.9 | 143 | Resistance | 1.97 | 38.2 |
| 39 | Control CRC | 1.82 | 34.7 | 144 | Resistance | 1.96 | 27.9 |
| 40 | Control CRC | 1.98 | 37.0 | 145 | Resistance | 2.05 | 20.6 |
| 41 | Control CRC | 1.83 | 47.7 | 146 | Resistance | 1.98 | 31.4 |
| 42 | Control CRC | 1.92 | 21.8 | 147 | Resistance | 1.80 | 40.1 |
| 43 | Control CRC | 1.85 | 47.5 | 148 | Resistance | 1.84 | 21.3 |
| 44 | Control CRC | 1.86 | 31.0 | 149 | Resistance | 1.80 | 41.2 |
| 45 | Control CRC | 2.06 | 23.9 | 150 | Resistance | 1.92 | 38.7 |
| 46 | Control CRC | 2.04 | 32.5 | 151 | Resistance | 1.83 | 33.9 |
| 47 | Control CRC | 1.88 | 49.1 | 152 | Resistance | 1.83 | 24.3 |
| 48 | Response | 1.80 | 50.3 | 153 | Resistance | 1.82 | 31.1 |
| 49 | Response | 2.10 | 32.5 | 154 | Resistance | 1.87 | 33.3 |
| 50 | Response | 1.93 | 29.9 | 155 | Resistance | 1.98 | 42.1 |
| 51 | Response | 1.93 | 28.8 | 156 | Resistance | 1.83 | 32.1 |
| 52 | Response | 1.95 | 22.0 | 157 | Resistance | 1.90 | 25.8 |
| 53 | Response | 1.89 | 22.9 | 158 | Resistance | 2.04 | 24.2 |
| 54 | Response | 1.83 | 19.0 | 159 | Resistance | 1.88 | 25.1 |
| 55 | Response | 1.85 | 43.7 | 160 | Resistance | 1.92 | 29.8 |
| 56 | Response | 1.84 | 42.8 | 161 | Resistance | 1.99 | 28.1 |
| 57 | Response | 2.00 | 42.8 | 162 | Resistance | 1.95 | 27.1 |
| 58 | Response | 1.83 | 22.4 | 163 | Resistance | 1.85 | 29.7 |
| 59 | Response | 1.88 | 39.5 | 164 | Resistance | 1.96 | 30.1 |
| 60 | Response | 1.90 | 48.5 | 165 | Resistance | 1.85 | 37.1 |
| 61 | Response | 2.05 | 44.0 | 166 | Resistance | 2.06 | 32.1 |
| 62 | Response | 2.06 | 29.2 | 167 | Resistance | 1.84 | 33.6 |
| 63 | Response | 1.82 | 23.6 | 168 | Resistance | 2.02 | 47.0 |
| 64 | Response | 2.09 | 22.9 | 169 | Resistance | 1.92 | 49.2 |
| 65 | Response | 2.05 | 36.1 | 170 | Resistance | 1.87 | 28.1 |
| 66 | Response | 1.97 | 26.6 | 171 | Resistance | 2.05 | 36.2 |
| 67 | Response | 2.10 | 38.3 | 172 | Resistance | 1.93 | 41.4 |
| 68 | Response | 1.85 | 19.5 | 173 | Resistance | 1.91 | 38.2 |
| 69 | Response | 2.09 | 49.7 | 174 | Resistance | 2.10 | 32.4 |
| 70 | Response | 1.97 | 32.8 | 175 | Resistance | 1.85 | 29.6 |
| 71 | Response | 2.03 | 51.8 | 176 | Resistance | 1.87 | 47.2 |
| 72 | Response | 2.03 | 50.8 | 177 | Resistance | 1.99 | 40.3 |
| 73 | Response | 2.10 | 51.8 | 178 | Resistance | 2.01 | 34.6 |
| 74 | Response | 2.08 | 43.3 | 179 | Resistance | 2.01 | 25.5 |
| 75 | Response | 1.82 | 32.1 | 180 | Resistance | 2.10 | 47.5 |
| 76 | Response | 1.92 | 23.7 | 181 | Resistance | 1.93 | 28.0 |
| 77 | Response | 1.82 | 40.3 | 182 | Resistance | 1.90 | 28.3 |
| 78 | Response | 2.01 | 45.0 | 183 | Resistance | 2.05 | 29.1 |
| 79 | Response | 2.06 | 29.0 | 184 | Resistance | 1.92 | 42.6 |
| 80 | Response | 2.09 | 23.0 | 185 | Resistance | 1.93 | 28.0 |
| 81 | Response | 1.93 | 41.5 | 186 | Resistance | 2.05 | 23.7 |
| 82 | Response | 1.93 | 33.7 | 187 | Resistance | 2.03 | 21.5 |
| 83 | Response | 1.97 | 36.1 | 188 | Resistance | 1.96 | 26.3 |
| 84 | Response | 2.02 | 19.5 | 189 | Resistance | 1.88 | 21.0 |
| 85 | Response | 1.81 | 38.3 | 190 | Resistance | 1.88 | 29.3 |
| 86 | Response | 2.05 | 26.7 | 191 | Resistance | 2.07 | 28.7 |
| 87 | Response | 2.03 | 50.5 | 192 | Resistance | 1.81 | 48.3 |
| 88 | Response | 1.93 | 24.0 | 193 | Resistance | 1.99 | 38.3 |
| 89 | Response | 2.08 | 24.2 | 194 | Resistance | 2.05 | 36.3 |
| 90 | Response | 1.92 | 32.4 | 195 | Resistance | 1.97 | 47.2 |
| 91 | Response | 1.93 | 49.5 | 196 | Resistance | 1.83 | 24.3 |
| 92 | Response | 2.06 | 38.4 | 197 | Resistance | 2.02 | 39.4 |
| 93 | Response | 1.94 | 35.0 | 198 | Resistance | 2.02 | 43.8 |
| 94 | Response | 2.00 | 52.4 | 199 | Resistance | 1.87 | 42.5 |
| 95 | Response | 2.10 | 49.8 | 200 | Resistance | 2.02 | 40.9 |
| 96 | Response | 1.85 | 46.4 | 201 | Resistance | 1.89 | 31.4 |
| 97 | Response | 1.89 | 27.5 | 202 | Resistance | 2.07 | 38.6 |
| 98 | Response | 1.90 | 27.7 | 203 | Resistance | 1.89 | 44.9 |
| 99 | Response | 2.06 | 22.9 | 204 | Resistance | 1.96 | 31.0 |
| 100 | Response | 1.91 | 43.6 | 205 | Resistance | 1.98 | 38.6 |
| 101 | Response | 1.96 | 44.7 | 206 | Resistance | 1.85 | 34.4 |
| 102 | Response | 2.06 | 39.3 | 207 | Resistance | 1.90 | 32.2 |
| 103 | Response | 2.06 | 41.1 | 208 | Resistance | 2.03 | 20.2 |
| 104 | Response | 1.95 | 19.7 | 209 | Resistance | 1.89 | 38.0 |
| 105 | Response | 2.07 | 27.6 | 210 | Resistance | 2.03 | 52.4 |

Supplemental Table 6

The relative concentrations of selected miRNAs confirmed by RT-qPCR in plasma exosomes from the resistance and response groups in the training set (n=12).

| **miRNA** | **Resistance** | **Response** | **Fold Change (Rs/Rp)** | ***P*-value** | **Results** |
| --- | --- | --- | --- | --- | --- |
| hsa-miR-184 | 2.69 (0.47) | 1.33 (0.29) | 2.02 | 0.014 | significant |
| hsa-miR-100-5p | 2.69 (0.29) | 1.25 (0.26) | 2.15 | 0.001 | significant |
| hsa-miR-10a-5p | 3.15 (0.51) | 1.35 (0.33) | 2.33 | 0.007 | significant |
| hsa-miR-92a-3p | 2.84 (0.47) | 1.26 (0.28) | 2.25 | 0.008 | significant |
| hsa-miR-101-3p | 1.04 (0.13) | 1.37 (0.22) | 0.76 | 0.21 | non-significant |
| hsa-let-7f-5p | 0.78 (0.12) | 1.15 (0.18) | 0.68 | 0.104 | non-significant |
| hsa-miR-144-3p |  |  |  |  | Ct value >35 |
| hsa-miR-30e-5p | 0.49 (0.08) | 1.06 (0.11) | 0.46 | 0.0004 | significant |
| hsa-miR-144-5p | 0.56 (0.06) | 1.17 (0.20) | 0.48 | 0.014 | significant |
| hsa-let-7i-5p | 0.46 (0.07) | 1.07 (0.12) | 0.43 | 0.0003 | significant |
| hsa-miR-4532 |  |  |  |  | Ct value >35 |
| has-miR-16 | 0.40 (0.05) | 1.03 (0.08) | 0.39 | 0.001 | significant |

miRNA data are expressed as mean (SE)

Supplemental Table 7

Comparison of the relative concentrations of 8 candidate miRNAs in plasma exosomes from control CRC patients, responsive patients and resistant patients by RT-qPCR.

| **miRNAs** | **Control CRC (n=47)** | **Response  (n=72)** | **Resistance (n=67)** | **Fold Change*** | ***P^a^*** | ***P^b^*** | ***P^c^*** |
| --- | --- | --- | --- | --- | --- | --- | --- |
| miR-10a-5p | 0.0088(0.0009) | 0.0086(0.0007) | 0.0121(0.0009) | 1.41 | 0.9904 | 0.0279 | 0.0074 |
| miR-184 | 0.1004(0.0084) | 0.1068(0.0010) | 0.1444(0.0103) | 1.35 | 0.9008 | 0.01 | 0.0145 |
| miR-100-5p | 0.0058(0.0005) | 0.0070(0.0005) | 0.0105(0.0006) | 1.50 | 0.3021 | <0.0001 | <0.0001 |
| miR-92a-3p | 0.5595(0.0495) | 0.3664(0.0289) | 0.8034(0.0622) | 2.19 | 0.0208 | 0.0027 | <0.0001 |
| miR-16 | 0.7528(0.0636) | 0.6349(0.0641) | 0.4059(0.0551) | 0.64 | 0.4001 | 0.0007 | 0.0166 |
| let-7i | 0.2343(0.0212) | 0.4449(0.02328) | 0.293(0.0198) | 0.66 | <0.0001 | 0.1785 | <0.0001 |
| miR-144-5p | 0.0056(0.0005) | 0.0115(0.0006) | 0.0072(0.0006) | 0.63 | <0.0001 | 0.1792 | <0.0001 |
| miR-30e-5p | 0.0914(0.0082) | 0.1769(0.0084) | 0.1081(0.0065) | 0.61 | <0.0001 | 0.3323 | <0.0001 |

miRNA data are mean (SE). *Fold Change= Resistance/Response. ^a^ Control CRC *vs* Response. ^b^ Control CRC *vs* Resistance. ^c^ Response *vs* Resistance.

Supplemental Table 8

| **Test Result Variable(s)** | **Response Group *vs* Resistance Group** | | | | |
| --- | --- | --- | --- | --- | --- |
|  | **Area** | **Std. Error** | **Asymptotic Sig.** | **Asymptotic 95%  Confidence Interval** | |
|  |  |  |  | **Lower Bound** | **Upper Bound** |
| miR-10a-5p | 0.637 | 0.047 | 0.004 | 0.545 | 0.729 |
| miR-184 | 0.659 | 0.047 | 0.001 | 0.568 | 0.751 |
| miR-100-5p | 0.694 | 0.045 | <0.0001 | 0.606 | 0.782 |
| miR-92a-3p | 0.791 | 0.037 | <0.0001 | 0.718 | 0.864 |
| miR-16 | 0.650 | 0.046 | 0.002 | 0.559 | 0.742 |
| has-let-7i | 0.721 | 0.043 | <0.0001 | 0.638 | 0.804 |
| miR-144-5p | 0.746 | 0.042 | <0.0001 | 0.664 | 0.827 |
| miR-30e-5p | 0.778 | 0.039 | <0.0001 | 0.702 | 0.854 |
| CEA | 0.542 | 0.049 | 0.391 | 0.446 | 0.638 |
| CA19-9 | 0.686 | 0.044 | 0.0002 | 0.599 | 0.773 |
| The panel^1^ | 0.825 | 0.037 | <0.0001 | 0.753 | 0.897 |
| The panel^2^ | 0.824 | 0.036 | <0.0001 | 0.752 | 0.896 |

The respective AUCs of 8 candidate miRNAs and CRC biomarkers in the ROC curve analysis of patients in the response phase and resistance phase.

^1^ The best combination of 6 miRNAs (miR-100, miR-92a, miR-16, let-7i, miR-144, and miR-30e).

2 The combination of 6 miRNAs as well as CEA and CA19-9.

Supplemental Table 9

The respective ORs of 6 candidate miRNAs in the univariate logistic regression model analysis of resistant patients and responsive patients.

| **miRNAs** | **B** | **Std.Error** | **OR** | **95% CI for OR** | | **P** |
| --- | --- | --- | --- | --- | --- | --- |
|  |  |  |  | **Lower** | **Upper** |  |
| miR-92 | 2.118 | 0.577 | 8.311 | 2.685 | 25.728 | <0.0001 |
| miR-100 | 1.59 | 0.592 | 4.904 | 1.536 | 15.651 | 0.007 |
| miR-16 | 1.502 | 0.596 | 4.491 | 1.397 | 14.437 | 0.012 |
| miR-30e | 1.832 | 0.584 | 6.245 | 1.989 | 19.604 | 0.002 |
| miR-144 | 2.185 | 0.575 | 8.886 | 2.878 | 27.44 | <0.0001 |
| let-7i | 1.906 | 0.582 | 6.729 | 2.153 | 21.036 | 0.001 |

B, regression coefficient; OR, odds ratio.

Supplemental Table 10

The results of Spearman’s rank correlations between 6 candidate plasma exosomal miRNAs and other variables in CRC patients. (n=139)

| **miRNA** | **Sex** | **Age** | **Location** | **Histological grade** | **TNM stage** | **Chemotherapy Program** |
| --- | --- | --- | --- | --- | --- | --- |
| miR-100 | 0.011 | -0.091 | 0.132 | -0.177* | 0.133 | -0.128 |
|  | *P=0.896* | *P=0.287* | *P=0.122* | *P=0.037* | *P=0.120* | *P=0.132* |
| miR-92a | -0.007 | 0.165 | 0.022 | -0.069 | -0.072 | -0.155 |
|  | *P=0.935* | *P=0.053* | *P=0.799* | *P=0.422* | *P=0.401* | *P=0.068* |
| miR-16 | -0.019 | -0.012 | -0.009 | 0.017 | 0.123 | -0.033 |
|  | *P=0.820* | *P=0.890* | *P=0.915* | *P=0.842* | *P=0.148* | *P=0.700* |
| miR-30e | 0.088 | -0.083 | -0.085 | 0.019 | -0.083 | -0.007 |
|  | *P=0.301* | *P=0.330* | *P=0.319* | *P=0.823* | *P=0.334* | *P=0.938* |
| miR-144-5p | 0.124 | -0.056 | 0.041 | 0.039 | -0.031 | -0.121 |
|  | *P=0.145* | *P=0.511* | *P=0.630* | *P=0.645* | *P=0.713* | *P=0.157* |
| let-7i | -0.047 | 0.014 | 0.005 | 0.045 | -0.021 | -0.058 |
|  | *P=0.584* | *P=0.873* | *P=0.953* | *P=0.601* | *P=0.808* | *P=0.500* |

Supplemental Table 11

The information of 4 genes selected from GO and KEGG analyses in upregulated group.

| **Name** | **Full Name** |
| --- | --- |
| **PTEN** | phosphatase and tensin homolog |
| **PIK3CB** | phosphatidylinositol-4,5-bisphosphate 3-kinase catalytic subunit beta |
| **PIK3CA** | phosphatidylinositol-4,5-bisphosphate 3-kinase catalytic subunit alpha |
| **PIK3R3** | phosphoinositide-3-kinase regulatory subunit 3 |

Supplemental Table 12

The information of 15 genes selected from GO and KEGG analyses in downregulated group.

| **Name** | **Full Name** |  |  |
| --- | --- | --- | --- |
| **KRAS** | KRAS proto-oncogene, GTPase | | |
| **FGF18** | Fibroblast growth factor 18 | |  |
| **FGF7** | Fibroblast growth factor 7 | |  |
| **FGF9** | Fibroblast growth factor 9 | |  |
| **FGF11** | Fibroblast growth factor 11 | |  |
| **FGF2** | Fibroblast growth factor 2 | |  |
| **AKT3** | Akt Serine/Threonine Kinase 3 | |  |
| **TP53** | Tumor Protein P53 |  |  |
| **FGFR1** | Fibroblast growth factor receptor 1 | | |
| **SOS1** | SOS Ras/Rac Guanine Nucleotide Exchange Factor 1 | | |
| **SOS2** | SOS Ras/Rac Guanine Nucleotide Exchange Factor 2 | | |
| **MAP2K1** | mitogen-activated protein kinase kinase 1 | | |
| **RAF1** | Raf-1 Proto-Oncogene | |  |
| **NRAS** | NRAS proto-oncogene, GTPase | | |
| **IKBKB** | inhibitor of nuclear factor kappa B kinase subunit beta | | |

Supplemental Table 13

The mature sequence and catalog ID (Applied Biosystems ) of the selected miRNAs.

| miRNA | Mature miRNA Sequence | Catalog ID |
| --- | --- | --- |
| miR-184 | UGGACGGAGAACUGAUAAGGGU | 000485 |
| miR-100 | AACCCGUAGAUCCGAACUUGUG | 000437 |
| miR-10a | UACCCUGUAGAUCCGAAUUUGUG | 000387 |
| miR-92a | UAUUGCACUUGUCCCGGCCUGU | 000431 |
| miR-16 | UAGCAGCACGUAAAUAUUGGCG | 000391 |
| miR-30e | UGUAAACAUCCUUGACUGGAAG | 002223 |
| miR-144-5p | GGAUAUCAUCAUAUACUGUAAG | 002148 |
| let-7i | UGAGGUAGUAGUUUGUGCUGUU | 002221 |
